# High-frequency spin torque oscillation in orthogonal magnetization disks with strong biquadratic magnetic coupling

**DOI:** 10.1038/s41598-023-30838-y

**Published:** 2023-03-03

**Authors:** C. Liu, Y. Kurokawa, N. Hashimoto, T. Tanaka, H. Yuasa

**Affiliations:** Graduate School and Faculty of Information Science and Electrical Engineering, Kyushu University, Fukuoka, 819-0395 Japan

**Keywords:** Electronic and spintronic devices, Applied physics, Magnetic properties and materials, Theory and computation

## Abstract

In this study, we numerically investigate the spin transfer torque oscillation (STO) in a magnetic orthogonal configuration by introducing a strong biquadratic magnetic coupling. The orthogonal configuration consists of top and bottom layers with in-plane and perpendicular magnetic anisotropy sandwiching a nonmagnetic spacer. The advantage of an orthogonal configuration is the high efficiency of spin transfer torque leading a high STO frequency; however, maintaining the STO in a wide range of electric current is challenging. By introducing biquadratic magnetic coupling into the orthogonal structure of FePt/spacer/Co_90_Fe_10_, Ni_80_Fe_20_ or Ni, we were able to expand the electric current region in which the stable STO is realized, resulting in a relatively high STO frequency. For example, approximately 50 GHz can be achieved in an Ni layer at a current density of 5.5 × 10^7^ A/cm^2^. In addition, we investigated two types of initial magnetic state: out-of-plane and in-plane magnetic saturation; this leads to a vortex and an in-plane magnetic domain structure after relaxation, respectively. The transient time before the stable STO was reduced to between 0.5 and 1.8 ns by changing the initial state from out-of-plane to in-plane.

## Introduction

Ever since it was discovered that magnetization dynamics can be controlled by spin transfer torque (STT)^1,2^, the approach has been widely studied and applied in data storage and memory applications, such as microwave-assisted magnetic recording (MAMR) in hard disk drives (HDDs) and magnetoresistive random access memory (MRAM)^3–8^. Recently, STO is also gathering expectations for neural networks applications^9–11^. While typical ferromagnetic transition metals show spin torque oscillation (STO) frequencies of several GHz^12–15^, antiferromagnets are presumed to have STO frequencies in the THz region; this assumption is in light of various theoretical studies, including those on magnetic resonance frequency^16–29^. However, usable STOs have never been achieved experimentally in antiferromagnets because of the strong exchange coupling between neighboring ions. Therefore, artificial magnetic structures such as synthetic antiferromagnetic coupling layers^30–32^ and quasi-antiferromagnets comprising biquadratic magnetic coupling^33,34^ have been proposed as an alternative to increase the STO frequency. On the other hand, an orthogonal magnetization multilayer comprising in-plane and perpendicular anisotropic magnetic layers was reported to have a high spin transfer efficiency that allows an STO to be excited easily, suggesting a spin transfer torque oscillator with high frequency and low power consumption^35–42^. The high efficiency of the STT originates from the small angle between the magnetizations of the two magnetic layers, which unfortunately reduces the current density range that shows a stable STO, considering magnetization switching occurs frequently. Therefore, a biquadratic magnetic coupling^33,34,43–55^ was introduced between the two magnetic layers in the orthogonal configuration to suppress complete magnetization switching, thereby improving the stability of the STO. Biquadratic magnetic coupling is an interlayer exchange interaction between two ferromagnetic (FM) layers separated by a thin spacer. In general, the magnetic coupling energy E is expanded to a higher-order equation by considering the quadratic term as E =  − *A*_12_**M**_1_∙**M**_2_ − *B*_12_ (**M**_1_∙**M**_2_)^2^, where **M**_1_ and **M**_2_ are the unit magnetizations in the first and second FM layers, respectively, and *A*_12_ and *B*_12_ are the bilinear and biquadratic coupling coefficients, respectively. *A*_12_ contributes to 0° or 180° magnetic coupling, whereas *B*_12_ contributes to + / − 90° magnetic coupling. Typically, + / − 90° coupling is realized under the conditions of |*A*_12_|< 2|*B*_12_| and *B*_12_ < 0, using a suitable layer as a spacer. The values of *A*_12_ and *B*_12_ depend on the spacer layer material and the magnetic material, respectively, and values of 0 erg/cm^2^ to –0.24 erg/cm^2^ for *A*_12_ and − 0.005 erg/cm^2^ to–2.0 erg/cm^2^ for *B*_12_ have been reported^33,34,43–55^. Previously, we reported that biquadratic coupling simply works as an effective field and increases the STO frequency in the quasi-antiferromagnet Co_90_Fe_10_^33,34^. However, the frequency was limited to 15 GHz under a realistic current density, using a multilayer with two in-plane magnetization layers. After that, we reported that a high frequency was obtained by applying biquadratic coupling to an orthogonal configuration, but there were still remained issues of frequency stability and long transient time^42^. In this study, we report a numerical experiment of STO behavior in an orthogonal magnetization configuration with biquadratic coupling. Additionally, we investigate the initial state to obtain a fast response with a short transient time before the stable STO occurs.

## Methods

### Calculation model

**Figure 1:**
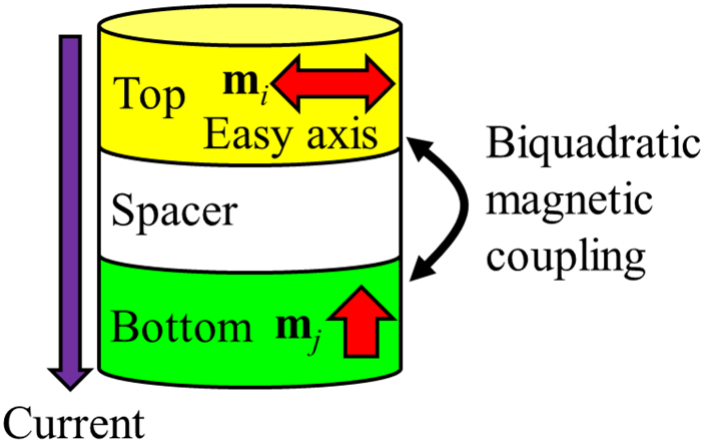
Calculation model with an orthogonal structure.

The magnetization dynamics were investigated by solving the Landau–Lifshitz–Gilbert (LLG) equation with an STT term, given by

1 $$\begin{array}{c}\frac{d\mathbf{m}}{dt}=-\gamma \left(\mathbf{m}\times {\mathbf{H}}_{\mathbf{e}\mathbf{f}\mathbf{f}}\right)+\alpha \left(\mathbf{m}\times \frac{d\mathbf{m}}{dt}\right)-\frac{g{\mu }_{B}Jp}{{M}_{s}{d}_{i}e}\left(\mathbf{m}\times \left({\mathbf{m}}_{\mathrm{p}}\times \mathbf{m}\right)\right)\end{array}$$

where **m** and ${\mathbf{m}}_{\mathrm{p}}$ are the normalized magnetization of the top free layer and bottom pinned layer, respectively, γ is the gyromagnetic ratio, αis the damping constant, $g$ is the gyromagnetic splitting factor, ${\mu }_{B}$ is the Bohr magneton, *J* is the current density, *p* is the polarity of current, *M*_s_ is the saturation magnetization of the top layer, *d*_*i*_ is the thickness of the top layer, and **H**_eff_ is the effective field, which consists of the following fields: **H**_eff_ = **H**_K_ + **H**_exf_ + **H**_ex_ + **H**_ST_ + **H**_bq_ + **H**_bl_, where **H**_K_ is the magnetic anisotropy field, **H**_exf_ is the external magnetic field, **H**_ex_ is the exchange coupling field determined by the exchange stiffness constant *A*, **H**_ST_ is the stray field that depends on the saturation magnetization of the magnetic material, **H**_bq_ is the biquadratic exchange field determined by the biquadratic coefficient *B*_12_, and **H**_bl_ is the bilinear exchange field determined by the bilinear coefficient *A*_12_. When the two ferromagnetic (FM) layers, *i* and *j*, have normalized magnetizations *m*_*i*_ and *m*_*j*_, respectively, the bilinear and biquadratic exchange fields **H**_bl_ and **H**_bq_ are given by^56^

$${\mathbf{H}}_{\mathrm{bl},i}=\frac{{A}_{12}}{{\mu }_{0}{{M}_{s}}_{,i}{d}_{i}}{\mathbf{m}}_{j}$$

and

2 $$\begin{array}{c}{\mathbf{H}}_{\mathrm{bq},i}=\frac{{2B}_{12}}{{{M}_{s}}_{,i}{d}_{i}}\left[\begin{array}{c}{m}_{i,x}{m}_{j,x}^{2}+{m}_{j,x}\left({m}_{i,y}{m}_{j,y}+{m}_{i,z}{m}_{j,z}\right)\\ {m}_{i,y}{m}_{j,y}^{2}+{m}_{j,y}\left({m}_{i,x}{m}_{j,x}+{m}_{i,z}{m}_{j,z}\right)\\ {m}_{i,z}{m}_{j,z}^{2}+{m}_{j,z}\left({m}_{i,x}{m}_{j,x}+{m}_{i,y}{m}_{j,y}\right)\end{array}\right]with\,i\ne j\end{array}$$

where *M*_*s,i*_ is the saturation magnetization of the magnetic material of the top layer. The *xy* plane and *z*-axis are the in-plane and perpendicular directions, respectively, as shown in Fig. 1.

In the orthogonal configuration, the top and bottom FM layers are assumed to be *i* and *j* with in-plane and perpendicular magnetic anisotropy, respectively. The bilinear exchange field **H**_bl,*i*_ is parallel to the magnetization **m**_*j*_, and the biquadratic exchange field **H**_bq,*i*_ becomes

3 $$\begin{array}{c}{\mathbf{H}}_{\mathrm{bq},i}=\frac{{2B}_{12}}{{{M}_{s}}_{,i}{d}_{i}}\left[\begin{array}{c}0\\ 0\\ {m}_{i,z}\end{array}\right]\end{array}$$

This indicates that **H**_bq_ is parallel to **m**_*j*_ for *B*_12_ > 0 and antiparallel to **m**_*j*_ for *B*_12_ < 0. Because the biquadratic magnetic coupling becomes apparent under the condition *B*_12_ < 0, the direction of **H**_bq_ is parallel or opposite to **m**_*j*_ when the biquadratic exchange magnetic coupling is realized. Here, the STT term is parallel to **m**_*j*_ in the LLG equation, so that the biquadratic exchange coupling **H**_bq_ is in the opposite direction to the STT. Considering that **H**_bq_ is dominant in the effective field **H**_eff_ as well as **H**_ST_, the biquadratic exchange coupling **H**_bq_ is important for suppressing the complete magnetization reversal from the in-plane to the perpendicular direction, resulting in a stable STO. The magnetic moment of the top layer is expected to exhibit a gyro motion around the z-axis.

In the proposed model, the orthogonal configuration consisted of FePt 2 nm/spacer 2 nm/Co_90_Fe_10_, Ni_80_Fe_20_, or Ni 2 nm. The device was disk-shaped with a diameter of 320 nm. The bottom layer of FePt had a perpendicular anisotropy, with **H**_k_ = 15,000 Oe and saturation magnetization *M*_*s*_ = 800 emu/cc^57,58^. The bottom FM layer (j) has the fixed magnetization by setting *m*_j,x_ = *m*_j,y_ = 0, and *m*_j,z_ = 1 and as the current flows through its huge **H**_k_ to maintain perpendicular magnetization. The top layer had an in-plane magnetic anisotropy: Co_90_Fe_10_ had **H**_k_ = 35 Oe, *M*_s_ = 1450 emu/cc^33^ and a damping constant $\mathrm{\alpha }$ of 0.035; Ni_80_Fe_20_ had **H**_k_ = 2 Oe, *M*_s_ = 825 emu/cc and a damping constant $\mathrm{\alpha }$ of 0.008^59^; and Ni had **H**_k_ = 2 Oe, *M*_s_ = 510 emu/cc and a damping constant $\mathrm{\alpha }$ of 0.0088^60^. The bilinear coefficient *A*_12_ was set as 0 for simplicity and the biquadratic coefficient *B*_12_ was set to − 0.6, where *B*_12_ was reported in the experimental value of the spacer Fe-O^33^. Both *A*_12_ and *B*_12_ are realistic values from reports so far^33,34,43–55^. The magnetic unit cell size was 5 nm × 5 nm × 2 nm, and 64 × 64 × 3 cells were calculated. The exchange stiffness constant *A* was 1 × 10^−6^ erg/cm and the gyromagnetic ratio *γ* was 1.76 × 10^7^ Oe^−1^ s^−1^. The calculation step *dt* was 10 fs.

In the first step, we calculated the magnetization relaxation after saturating the magnetization along the *z*- and *y*-axes. Then, the current was made to flow through the layers, from the top to the bottom layer. Because the required current density for the STO generally depends on the saturation magnetization *M*_s_, we varied the current density from 8.0 × 10^7^ A/cm^2^ to 20.0 × 10^7^ A/cm^2^ for the Co_90_Fe_10_ top layer, and from 0.5 × 10^7^ A/cm^2^ to 6.0 × 10^7^ A/cm^2^ for the Ni_80_Fe_20_ and Ni top layers.

## Results and discussion

### Static and dynamic properties with *z*-axis magnetization saturation in initial state

**Figure 2:**
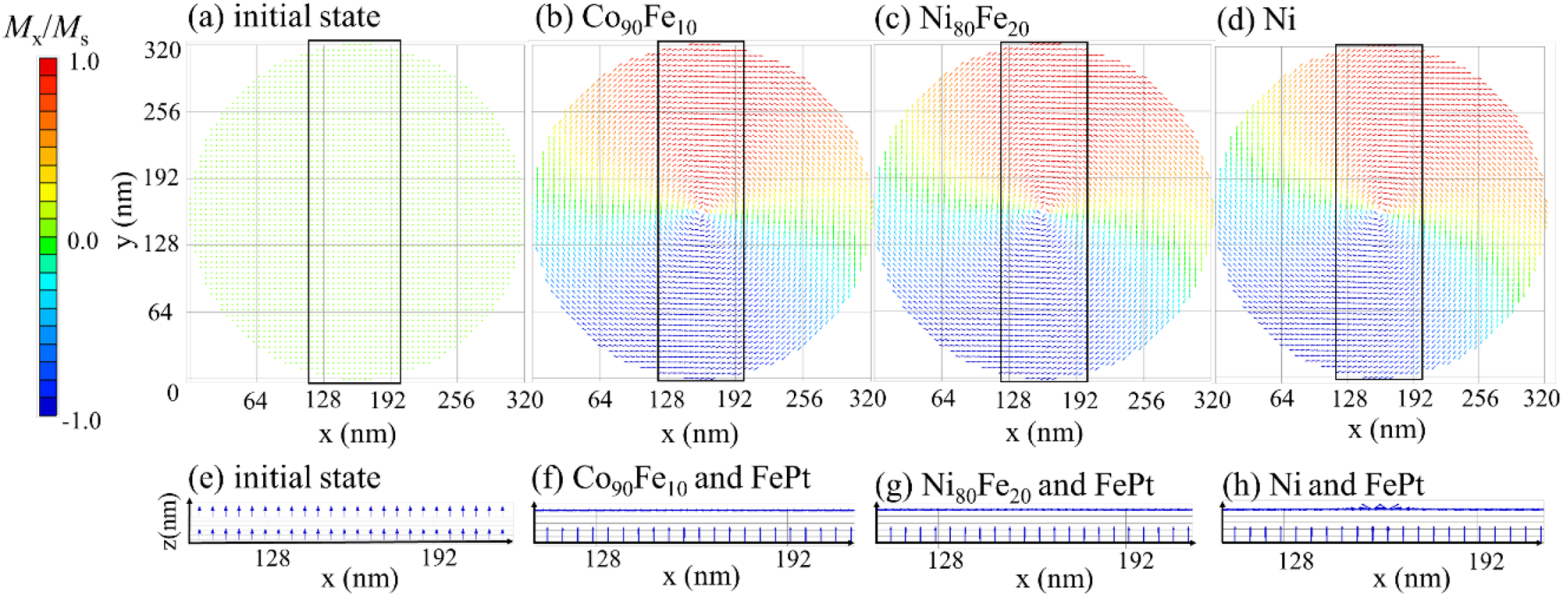
[Top] Top views of the magnetization configuration of the top layers for (**a**) the initial state of* z*-axis magnetization saturation for Co_90_Fe_10_ 2 nm, (**b**) the relaxed state of Co_90_Fe_10_ 2 nm, (**c**) the relaxed state of Ni_80_Fe_20_ 2 nm, and (**d**) the relaxed state of Ni 2 nm when *A*_12_ = 0 and *B*_12_ =  − 0.6. In the initial state, the configuration does not depend on the material of the top layer. [Bottom] Side views of the magnetization configuration in bottom FePt 2 nm/spacer 2 nm/top layer 2 nm when the top layer is (**a**) the Co_90_Fe_10_ 2 nm in the initial state, (**b**) the relaxed state of Co_90_Fe_10_ 2 nm, (**c**) the relaxed state of Ni_80_Fe_20_ 2 nm, and (**d**) the relaxed state of Ni 2 nm, corresponding to the enclosed area in (**e**–**h**). The top and bottom arrows show the magnetization of the top and bottom layers, respectively. Magnetic moments in the *y*-direction are superimposed on each *x* position.

First, we calculated the magnetization relaxation subject to the initial condition that the magnetization of the top layer was out-of-plane, *i. e.,* in the *z*-axis, as shown in Fig. 2a. Figure 2b–d show the calculated magnetization maps after relaxation in the top layers of Co_90_Fe_10_, Ni_80_Fe_20_, and Ni, respectively. Figure 2e–g show the side views of the magnetization configuration of the top and bottom layers, which correspond to the areas surrounded by black lines in Fig. 2b–d, where all magnetizations in the *y*-direction were superimposed on each *x* position. Neither external fields nor electrical currents were applied here. In this case, the exchange stiffness energy acted equally on the magnetizations of adjacent cells, causing the relaxed state of the top layer to become a vortex state, as shown in Fig. 2b–d. The bottom layer of FePt exhibited perpendicular magnetization owing to the sufficiently high perpendicular anisotropy energy.

**Figure 3:**
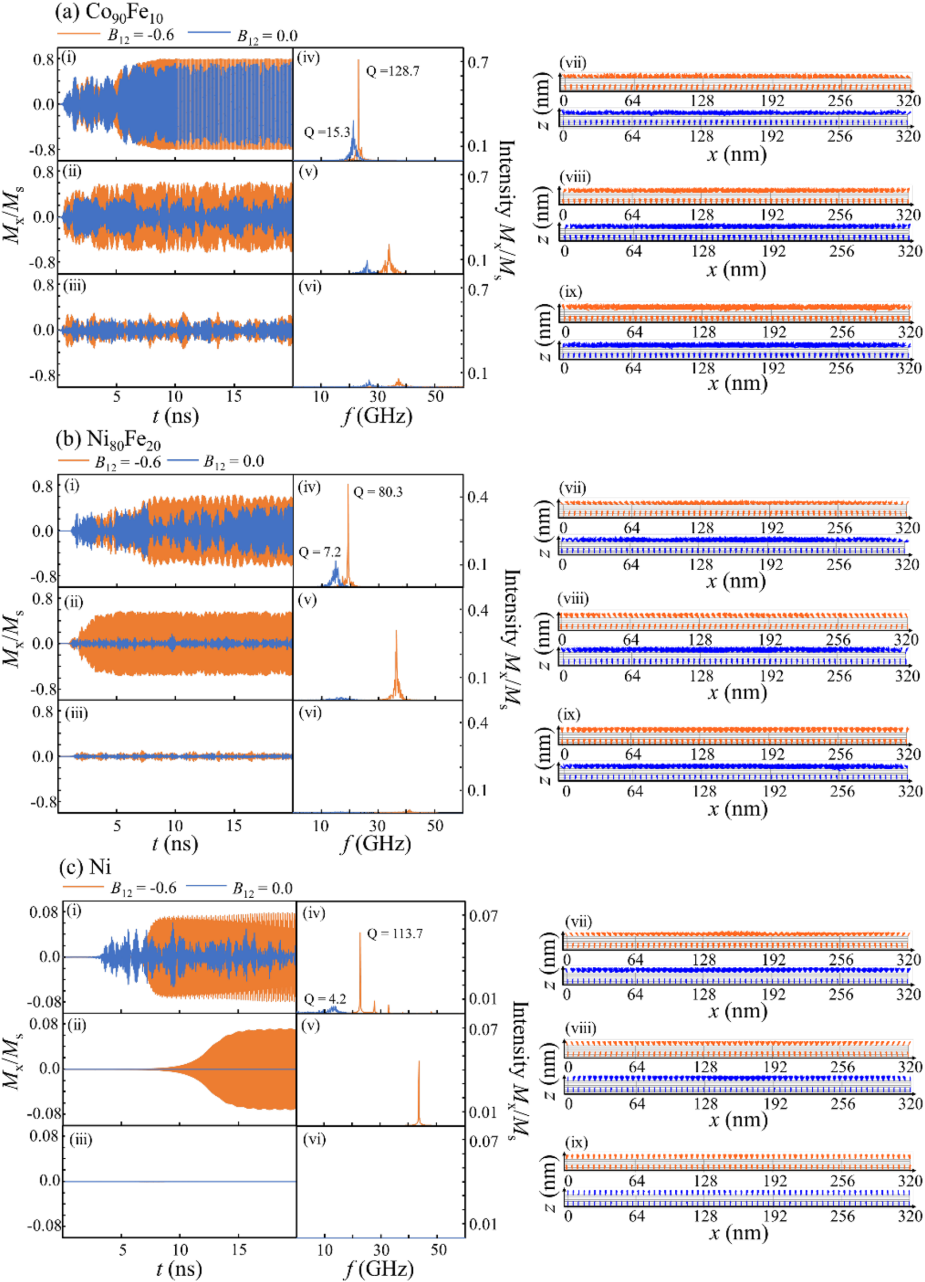
STO performances for the top layer of (**a**) Co_90_Fe_10_, (**b**) Ni_80_Fe_20_, and (**c**) Ni in the orthogonal configuration with an out-of-plane initial state, namely the *z*-axis. (i–iii) Time-domain magnetization precessions *M*_*x*_/*M*_s_, (iv–vi) Fast Fourier transform spectra, and (vii)-(ix) side views of magnetization configuration. The light blue and orange lines denote the results for *B*_12_ = 0.0 and *B*_12_ =  − 0.6, respectively. For the time-domain magnetization precessions *M*_*x*_/*M*_*s*_, the electrical current density was (i) 10 × 10^7^ A/cm^2^, (ii) 15 × 10^7^ A/cm^2^, and (iii) 20 × 10^7^ A/cm^2^ for Co_90_Fe_10_, and (i) 1.5 × 10^7^ A/cm^2^, (ii) 3.5 × 10^7^ A/cm^2^, and (iii) 6.0 × 10^7^ A/cm^2^ for Ni_80_Fe_20_ and Ni.

In the next step, the current flowed through the device from the top to the bottom layers. Figure 3a–c show the [(i–iii)] time-domain magnetization precession, [(iv)-(vi)] Fast Fourier transform (FFT) spectra, and [(vii)-(ix)] side views of the magnetization configuration of the top and bottom layers at 20 ns after the current flow, when *B*_12_ is 0.0 or -0.6 for (a) Co_90_Fe_10_, (b) Ni_80_Fe_20_, and (c) Ni, respectively. The electrical current density was (i) 10 × 10^7^ A/cm^2^, (ii) 15 × 10^7^ A/cm^2^, and (iii) 20 × 10^7^ A/cm^2^ for Co_90_Fe_10_ in Fig. 3a, and (i) 1.5 × 10^7^ A/cm^2^, (ii) 3.5 × 10^7^ A/cm^2^, (iii) and 6.0 × 10^7^ A/cm^2^ for Ni_80_Fe_20_ and Ni in Fig. 3b,c. Furthermore, we tuned the current density to obtain the stable STO considering this depends on the top layer materials, as previously discussed. The calculated data under the wide current density, including Fig. 3, are also shown in Fig. S1, S2 and S3 in Supplementary material.

We now focus on the time domains when the current density is increased, as shown in Fig. 3b,c [(i)-(iii)]. For all top layers, an STO was observed with a *B*_12_ of either 0.0 or -0.6 at a relatively low current density, shown in (i). However, as shown in (ii) and (iii), as the current density increased, the stable STO with an almost immutable *M*_x_/*M*_s_ is lost; in other words, the degradation of *M*_x_/*M*_s_ when *B*_12_ = 0.0 is larger compared to when *B*_12_ = -0.6. This means that by introducing biquadratic magnetic coupling, we can expand the electric current region where the stable STO is realized. Therefore, the current density can be increased, which results in a high frequency in the STO. Additionally, the FFT spectra in (iv)-(vi) show that the STO frequency is higher for *B*_12_ = -0.6 than *B*_12_ = 0.0, even for the same current density. We also calculate the quality factor Q. The FFT curve was fitted with a Gaussian distribution while changing the standard deviation σ. The quality factor was derived from the FWHM of the fitting curve when the residual error between Gaussian distribution curve and FFT curve is minimum. Since the quality factor Q increased by introducing *B*_12_, the extrinsic disturbance decreased and the ideal frequency became evident, resulting a high STO frequency. This was supported by the side views of the magnetization in (vii–ix). The variation of magnetization precession angle of the top layer is larger when *B*_12_ = 0.0 than *B*_12_ = −0.6 for the same current density in the same top layer materials. Moreover, the variation of the precession angle led to a poor Q factor. The detail comparison of angle variation is shown in Supplementary material.

Changing the top layer from Co_90_Fe_10_ and Ni_80_Fe_20_ to Ni, *M*_s_ becomes a small value and the current density required to realize the STO can be reduced. The combination of biquadratic coupling and *M*_s_ reduction is another possible approach for improving the frequency, as reported in Ref.^61^.

In conclusion, we revealed the STO characteristics in an orthogonal magnetization sample with biquadratic coupling. We found that the transient time until the stable STO was approximately 5–15 ns, which is too long for oscillators to be ignored. Therefore, in the following, we find the optimal initial state for a short transient time.

### Static and dynamic properties with y-axis magnetization saturation in initial state

**Figure 4:**
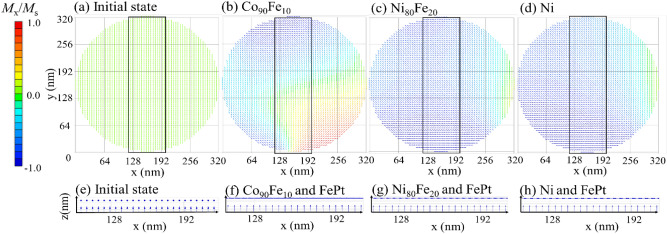
[Top] Top views of the magnetization configuration of the top layers for (**a**) the initial state of* y*-axis magnetization saturation for Co_90_Fe_10_ 2 nm, (**b**) the relaxed state of Co_90_Fe_10_ 2 nm, (**c**) the relaxed state of Ni_80_Fe_20_ 2 nm, and (d) the relaxed state of Ni 2 nm when *A*_12_ = 0 and *B*_12_ =  − 0.6. In the initial state, the configuration does not depend on the material of the top layer. [Bottom] Side views of magnetization configuration in bottom FePt 2 nm/spacer 2 nm/top layer 2 nm when the top layer is (**a**) the Co_90_Fe_10_ 2 nm in the initial state, (**b**) the relaxed state of Co_90_Fe_10_ 2 nm, (**c**) the relaxed state of Ni_80_Fe_20_ 2 nm, and (**d**) the relaxed state of Ni 2 nm, corresponding to the enclosed area in (**e**–**h**). The top and bottom arrows show the magnetization of the top and bottom layers, respectively. Magnetic moments in the *y*-direction are superimposed on each *x* position.

 Figure 4a shows the initial magnetization state before relaxation, where the magnetization of the top layer is saturated in-plane, *i. e.,* the *y*-axis, by a sufficient external field. The calculated magnetization maps after relaxation in the top layers, Co_90_Fe_10_, Ni_80_Fe_20_, and Ni, are shown in Fig. 4b–d, respectively, when neither external fields nor electrical currents were applied. All layers exhibited typical magnetic domains with in-plane magnetization, i.e., a single domain or onion domain, with a domain wall where the magnetization gradually rotated. Technically, only the Co_90_Fe_10_ top layer had an onion domain; the Ni_80_Fe_20_ and Ni top layers exhibited a single domain with magnetization at an angle of 45° from the *x*-axis owing to the difference in the magnetostatic energy that varied with the saturation of the magnetization. On comparing Figs. 2 and 4, we see that the relaxed top layer magnetization structure depends on whether the magnetization was initially saturated on the *z*-axis or *y*-axis. The side views of the magnetization configuration of the top and bottom layers are shown in Fig. 4e–g. The magnetization of the bottom layers of the three materials were along the *z*-axis, and the magnetization of the top layers were oriented along the *xy*-plane, forming an orthogonal configuration. The orthogonal structure was regularly obtained regardless of the initial state prior to relaxation.

**Figure 5:**
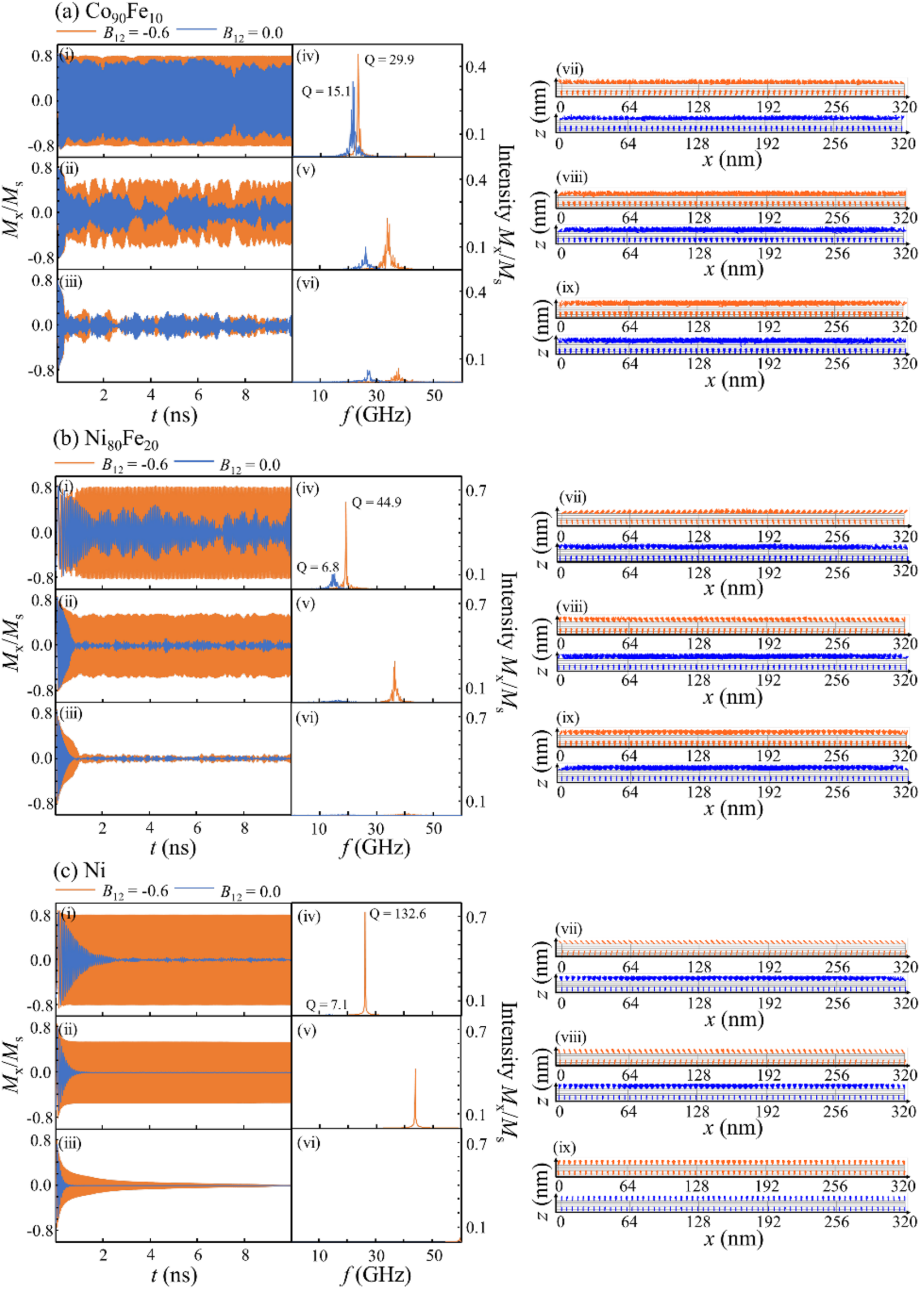
STO performances for the top layer of (**a**) Co_90_Fe_10_, (**b)** Ni_80_Fe_20_, and (**c**) Ni in the orthogonal configuration with an out-of-plane initial state, namely the *y*-axis. (i–ii) Time-domain magnetization precessions *M*_*x*_/*M*_s_, (iv–vi) the Fast Fourier transform spectra, and (vii–ix) side views of magnetization configuration. The light blue and orange lines denote the results for *B*_12_ = 0.0 and *B*_12_ =  − 0.6, respectively. For the time-domain magnetization precessions *M*_*x*_/*M*_*s*_, the electrical current density was (i) 10 × 10^7^ A/cm^2^, (ii) 15 × 10^7^ A/cm^2^, and (iii) 20 × 10^7^ A/cm^2^ for Co_90_Fe_10_, and (i) 1.5 × 10^7^ A/cm^2^, (ii) 3.5 × 10^7^ A/cm^2^, and (iii) 6.0 × 10^7^ A/cm^2^ for Ni_80_Fe_20_ and Ni.

Next, we considered the dynamics when the current flows through the device from the top to the bottom layers, similar to the case of initial state with *z*-axis magnetization saturation in the previous section. Figure 5a–c show the [(i–iii)] time-domain magnetization precession, [(iv–vi)] Fast Fourier transform (FFT) spectra, and [(vii–ix)] side view snapshots of the magnetization configuration of the top and bottom layers at 10 ns after the current flow, with *B*_12_ is 0.0 or −0.6, for (a) Co_90_Fe_10_, (b) Ni_80_Fe_20_, and (c) Ni. The electrical current densities were set as the same values as those for the *z*-axis saturation at the initial state in the previous section. The calculated data under the wide current density, including Fig. 5, are also shown in Fig. S4, S5 and S6 in Supplementary material. The common tendencies were obtained in both initial states of *y*-axis and *z*-axis saturation; the sample with *B*_12_ = −0.6 exhibited a more stable STO with an almost immutable *M*_x_/*M*_s_, a higher quality factor Q, and higher frequency than that with *B*_12_ = 0.0. We found that the biquadratic coupling is effective for increasing the frequency, regardless of the initial state.

However, there is a clear difference in the transient time between the initial state with *y*-axis and *z*-axis magnetization saturation. For the initial state with *z*-axis saturation magnetization, the transient times were 5.5, 8.9, and 11.2 ns for Co_90_Fe_10_, Ni_80_Fe_20_, and Ni, respectively, as shown in Fig. 3. In contrast, for the initial state with *y*-axis saturation magnetization, the transient times were 0.7, 1.2, and 1.0 ns for Co_90_Fe_10_, Ni_80_Fe_20_, and Ni, respectively, as shown in Fig. 5. Therefore, the transient time was significantly reduced by changing the initial magnetization state. As an example, we have provided a video of the magnetic moment dynamics for the Ni layer under 3.0 × 10^7^ A/cm^2^ for the initial states of *z*-axis and *y*-axis saturation (see the Supplementary material).

Notably, we can see a long period fluctuation in the time domain of the STO in Figs. 3a–c and 5a and b. This explains the overlapping of several STO modes, as shown in the [(iv–vi)] FFT spectra and [(vii–ix)] side views of the magnetization. For the Ni top layer only, there was one peak in the FFT and the magnetization amplitude was relatively uniform because of its small *M*_s_.

Once the STO was obtained, the effect of the initial state on the STO frequency was found to be insignificant. However, for Ni only, the magnitude of *M*_x_/*M*_s_ was increased from 0.084 to 0.800 by changing the initial state from *z*-axis saturation to *y*-axis saturation, as shown in Figs. 3c and 5c. This indicates that the initial magnetization state affects the transient time as well as the STO intensity when the top layer is Ni. To determine the reason for the large change in *M*_x_/*M*_s_ only for Ni, we compared the side view snapshots of the magnetization between the two different the initial states in Figs. 3c and 5c. In Fig. 3c, the magnetization directions of the top Ni layer disperse in the *xy*-plane, which indicates that the trajectory of magnetization is not uniform in the film, which decreases the total intensity *M*_x_/*M*_s_. On the other hand, as shown in Fig. 5c, the magnetization directions of the top layer maintain the same direction, leading to a large total intensity *M*_x_/*M*_s_. We observed that the magnetization directions disperse in the *xy*-plane, but the* z* component of the total magnetization does not depend on the initial magnetization state, which will be explained in the followings.

### Effect of initial state and biquadratic coupling on the STO

**Figure 6:**
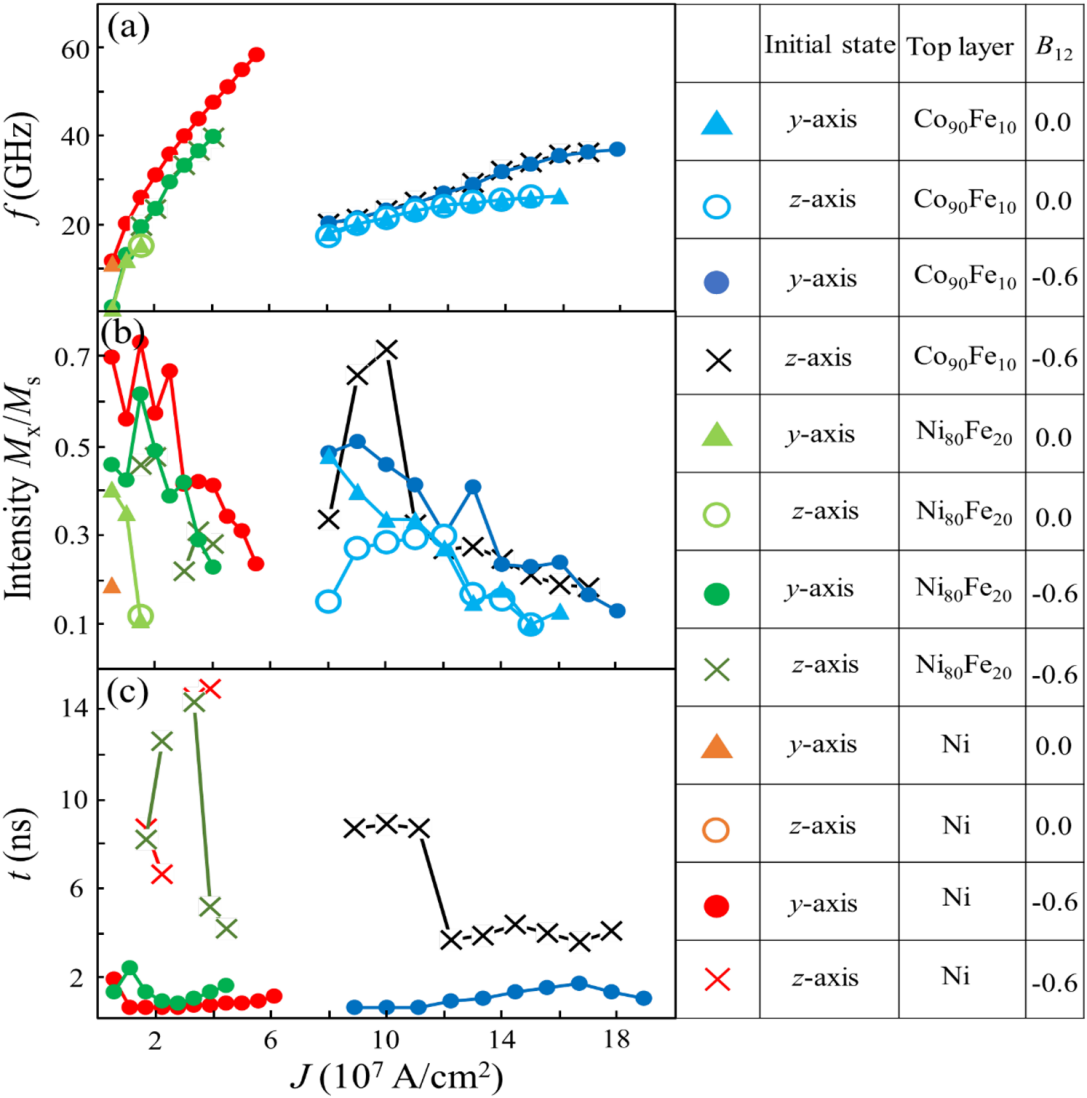
Current density dependences of (**a**) frequency, (**b**) intensity *M*_*x*_/*M*_s_, and (**c**) transient time in an orthogonal configuration. The solid circles and triangles denote the calculated results for the initial states of* y*-axis saturation when *B*_12_ is −0.6 or 0.0, respectively. The open squares and diamonds denote the calculated results for the initial states of* z*-axis saturation when *B*_12_ is −0.6 or 0.0, respectively.

Figure 6 shows the STO frequency, intensity *M*_x_/*M*_s_, and transient time as a function of the current density for the initial states of *z*-axis and *y*-axis saturation. The *M*_x_/*M*_s_ intensity over 0.1 is plotted. The solid circle and triangle denote the results for the initial states of* y*-axis saturation when *B*_12_ is −0.6 and 0.0, respectively. The open square and diamond denote the calculated results for the initial states of* z*-axis saturation when *B*_12_ is −0.6 and 0.0, respectively. In both cases, the critical current density for realizing the STO decreases and the frequency increases by changing the top layer from Co_90_Fe_10_ to Ni_80_Fe_20_ and Ni, depending on their *M*_s_. Provided the saturation magnetization does not change, the intensity *M*_x_/*M*_s_ decreases with an increase in current density. Whether the top layer is Ni, Ni_80_Fe_20_, or Co_90_Fe_10_, the current density region realizing the stable STO is widened by using *B*_12_ = −0.6 instead of *B*_12_ = 0. In particular, the biquadratic coupling effect is most obvious in the top layer of Ni, whose saturation magnetization is small when the initial state is *y*-axis saturation. There appears to be no significant difference in the frequencies and intensity of *M*_x_/*M*_s_ dependencies on the current density between the two initial states, except for Ni, which is consistent with the comparison results between Figs. 3c and 5c. In contrast to the frequency and intensity, a large discrepancy is observed in the transient times between the two initial states, as shown in Fig. 6c. Regardless of the current flow in any of the top layers, the initial state with *y*-axis saturation demonstrated a much smaller transient time than in the initial state with *z*-axis saturation. This tendency is particularly pronounced in the Ni_80_Fe_20_ and Ni top layers with an *M*_s_ smaller than that of Co_90_Fe_10_. We noted that the transient time did not change as the current density changed for an initial state of *y*-axis saturation; however, it varied widely for an initial state of *z*-axis saturation. This large variation in the transient time can be attributed to the dispersion of the magnetization directions in the *xy*-plane, as shown in the side views.

Finally, to confirm the effect of biquadratic coupling on STO performance, we compare the frequency, intensity, and transient time between the system with and without biquadratic coupling, namely between *B*_12_ = -0.6 and *B*_12_ = 0, highlighted by solid triangles and open diamonds in Fig. 6, respectively. The biquadratic coupling clearly increases the STO frequency for the same current density. However, more importantly, the current density region for which the STO was realized was expanded by the biquadratic coupling, resulting in the possibility that the maximum value of the frequency could be increased.

## Conclusion

In summary, we carried out a numerical investigation on spin torque oscillation in the magnetic multilayer FePt/spacer/Co_90_Fe_10_, Ni_80_Fe_20_, or Ni with an orthogonal magnetization configuration by introducing biquadratic magnetic coupling. The frequency and current density region were increased by introducing the biquadratic coupling, leading to 37 GHz, 40 GHz, and 58 GHz in the Co_90_Fe_10_, Ni_80_Fe_20_ and Ni layer at a current density of 18 × 10^7^ A/cm^2^, 4 × 10^7^ A/cm^2^, and 5.5 × 10^7^ A/cm^2^, respectively. In addition, we determined that two types of initial magnetic state, *z*-axis saturation and *y*-axis saturation, result in a vortex structure and in-plane onion magnetic structure after relaxation, respectively. The transient time before the stable STO was reduced from 0.5 to 1.8 ns by changing the initial state from *z*-axis saturation to *y*-axis saturation. The vortex structure is more stable than the onion structure, which is considered to be the reason for the difference in the transient time. By combining the orthogonal configuration, the biquadratic magnetic coupling, and the initial state with in-plane magnetic saturation, a stable STO with several tens of GHz frequency was obtained, which is advantageous for STO applications.

## Supplementary Information

Supplementary Information 1.

Supplementary Video 1.

Supplementary Video 2.

Supplementary Information 2.

## Data Availability

All data generated or analyzed during this study are included in this published article and its Supplementary Information files. The datasets used and/or analyzed during the current study available from the corresponding author on reasonable request.
