# Supplementary material for: High-frequency spin torque oscillation in orthogonal magnetization disks with strong biquadratic magnetic coupling
Source: Sci Rep. 2023 Mar 3;13:3631. doi: 10.1038/s41598-023-30838-y (PMC9984381; doi:10.1038/s41598-023-30838-y)
Supplement: Supplementary file 1 — Supplementary Information 1. [file 41598_2023_30838_MOESM1_ESM.pdf]

High Frequency Spin Torque Oscillation in Orthogonal Magnetization Disks with Strong Biquadratic Magnetic Coupling

*C. Liu, Y. Kurokawa, N. Hashimoto, T. Tanaka and †H. Yuasa

Graduate School and Faculty of Information Science and Electrical Engineering Kyushu University, Fukuoka 819-0395, Japan

*E-mail: *liu@mag.kyushu-u.ac.jp, †hiromi.yuasa@ed.kyushu-u.ac.jp

1. STO behaviors for various conditions

In order to confirm the current density limit realizing the stable magnetization oscillation, we calculated the STO properties with systematically and widely changing the current density for the top layer $Co_{90}Fe_{10}$, $Ni_{80}Fe_{20}$, and Ni in the orthogonal configuration where the initial state is out of plane (*z*-axis) and in-plane (*y*-axis). The calculation conditions are shown in Table S1. As well as Fig. 2 and Fig. 4, the time-domain magnetization precessions M_x/M_s, the Fast Fourier transform spectra, and side views of magnetization configuration are shown in Figs S1 to S6. Light blue and orange plot denotes the result for B_{12}=0.0 and B_{12}=−0.6. In addition, the top views of magnetization in the same condition as those in the side view, since the magnetization distribution is a cause of degradation of STO and quality factor. B_{12} = 0 and B_{12} = -0.6 but also show the distribution of the direction of each magnetization.

Table S1 Calculation conditions in Fig. S1-S12.

Fig.	View	Top layer	Magnetization in initial state	Current density (A/cm^2)
S1	Side view	$Co_{90}Fe_{10}$	out of plane (z axis)	8.0×10^7 ~ 20.0×10^7
S7	Top view			
S2	Side view	$Ni_{80}Fe_{20}$		0.5×10^7 ~ 6.0×10^7
S8	Top view			
S3	Side view	Ni		0.5×10^7 ~ 6.0×10^7
S9	Top view			
S4	Side view	$Co_{90}Fe_{10}$	in-plane (y axis)	8.0×10^7 ~20.0×10^7
S10	Top view			
S5	Side view	$Ni_{80}Fe_{20}$		0.5×10^7 ~ 6.0×10^7
S11	Top view			
S6	Side view	Ni		0.5×10^7 ~ 6.0×10^7
S12	Top view			

1. $Co_{90}Fe_{10}$ (z axis)

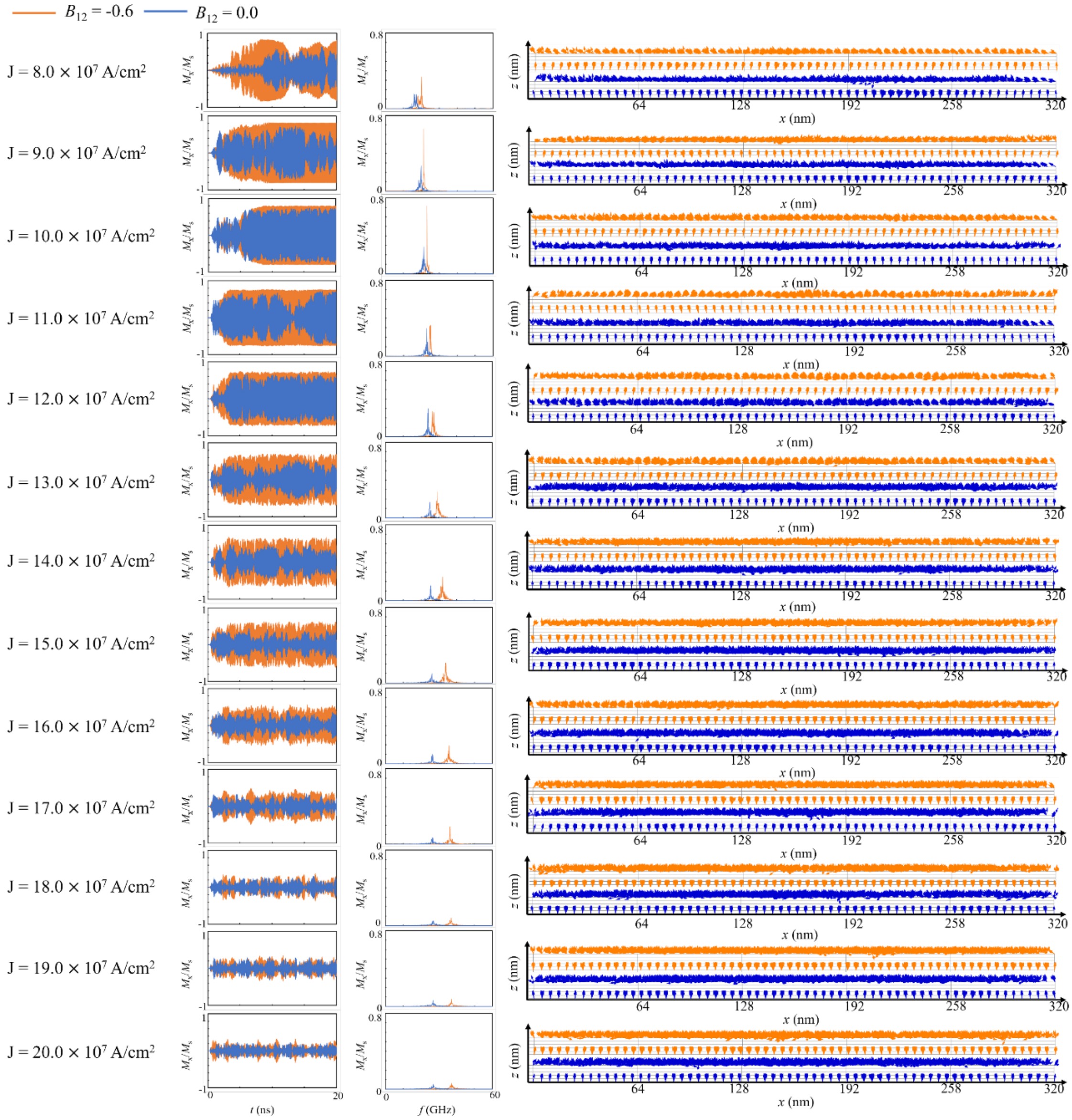

Fig. S1 STO performances for the top layer $Co_{90}Fe_{10}$ in the orthogonal configuration where the initial state is out of plane, namely z-axis. The electrical current density was varied from 8×10^7 A/cm^2 to 20×10^7 A/cm^2.

2. $Ni_{80}Fe_{20}$ (z axis)

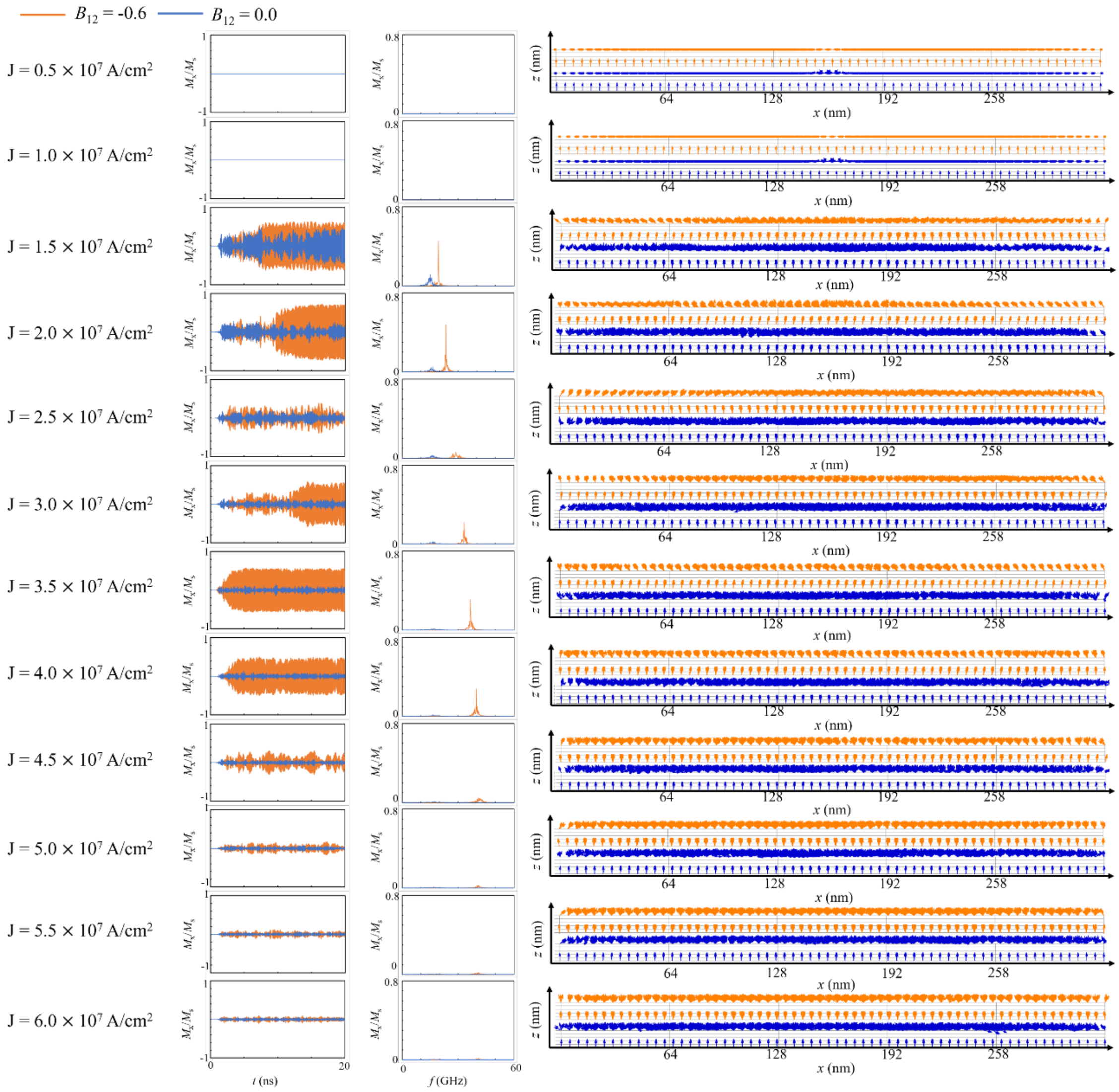

Fig. S2 STO performances for the top layer $Ni_{80}Fe_{20}$ in the orthogonal configuration where the initial state is out of plane, namely z-axis. The electrical current density was varied from 0.5×10^{7} A/cm^{2} to 6.0×10^{7} A/cm^{2}.

3. Ni (z axis)

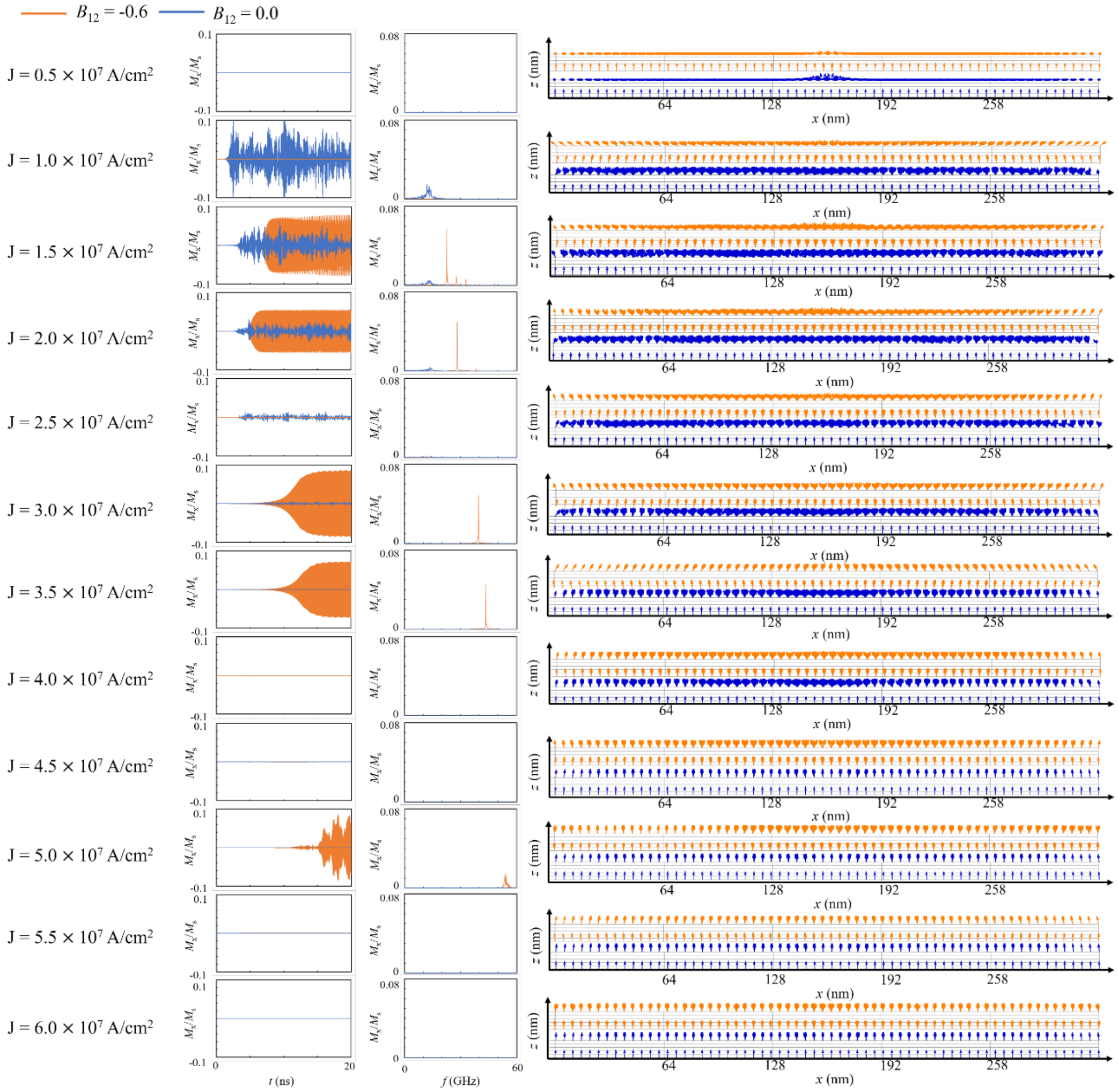

Fig. S3 STO performances for the top layer Ni in the orthogonal configuration where the initial state is out of plane, namely z-axis. The electrical current density was varied from 0.5×10^7 A/cm^2 to 6.0×10^7 A/cm^2.

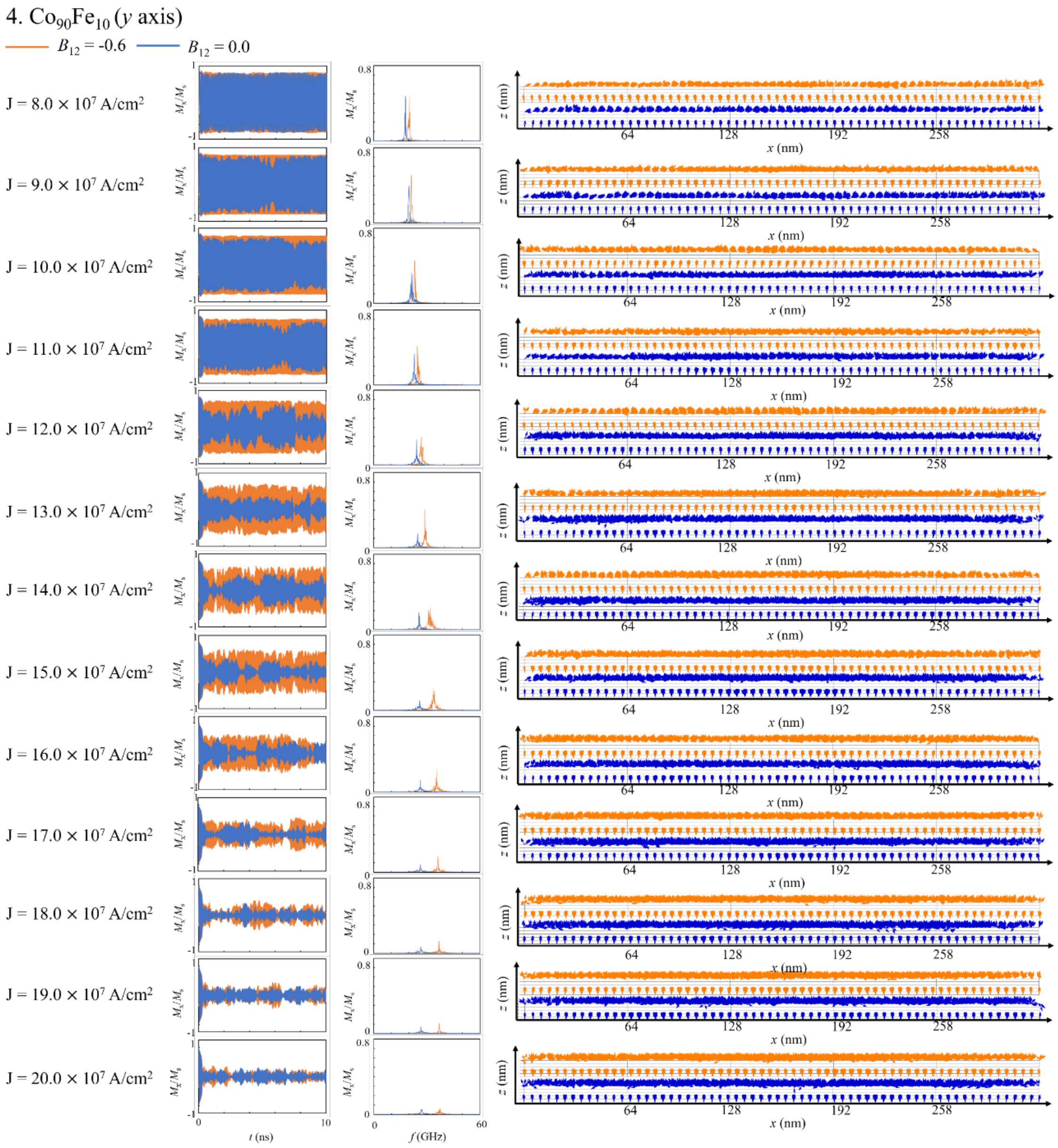

Fig. S4 STO performances for the top layer $Co_{90}Fe_{10}$ in the orthogonal configuration where the initial state is in- plane, namely *y*-axis. The electrical current density was varied from 8×10^7 A/cm^2 to 20×10^7 A/cm^2.

5. $Ni_{80}Fe_{20}$ (y axis)

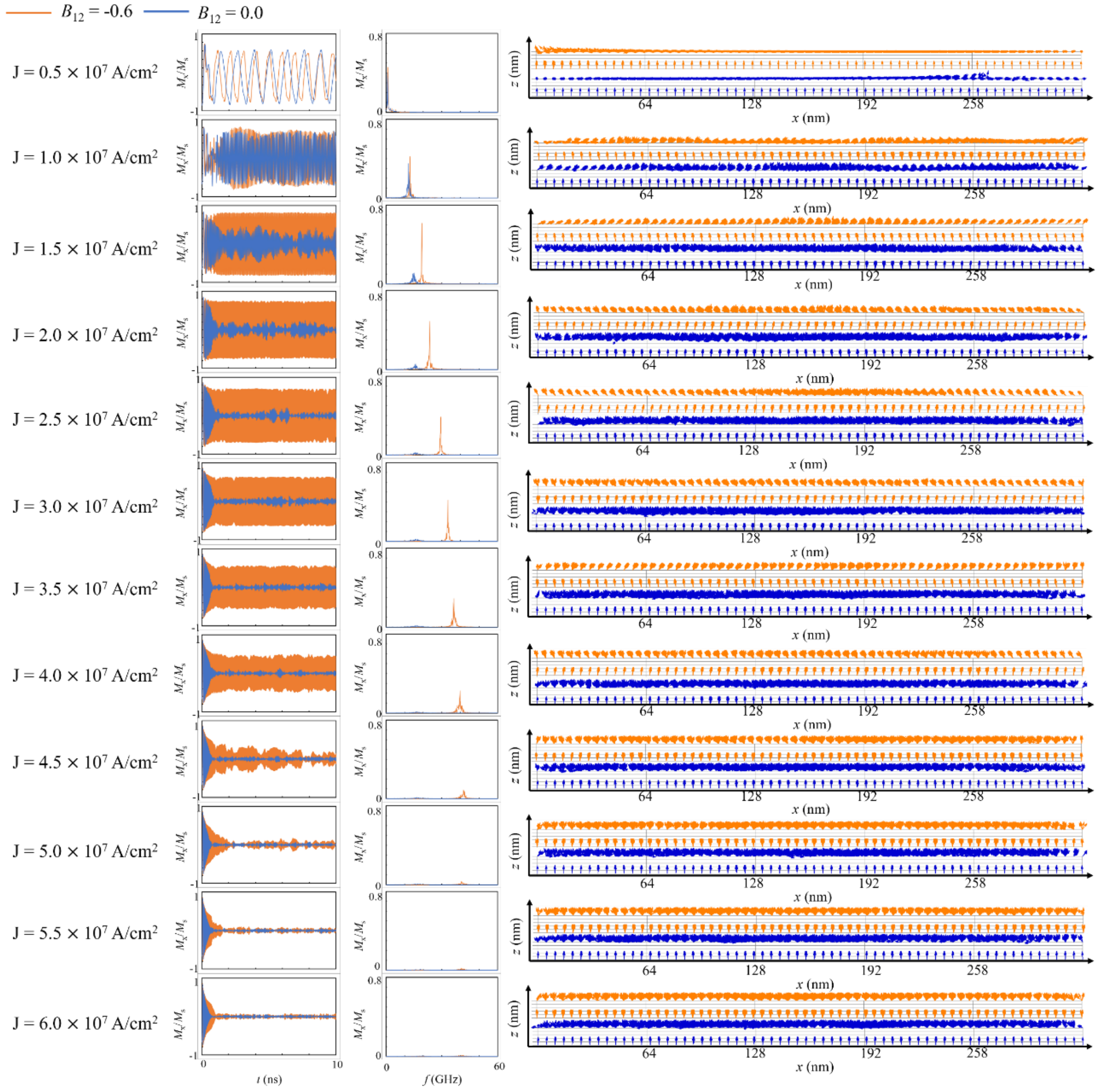

Fig. S5 STO performances for the top layer $Ni_{80}Fe_{20}$ in the orthogonal configuration where the initial state is in-plane, namely y-axis. The electrical current density was varied from 0.5×10^7 A/cm^2 to 6.0×10^7 A/cm^2.

6. Ni (y axis)

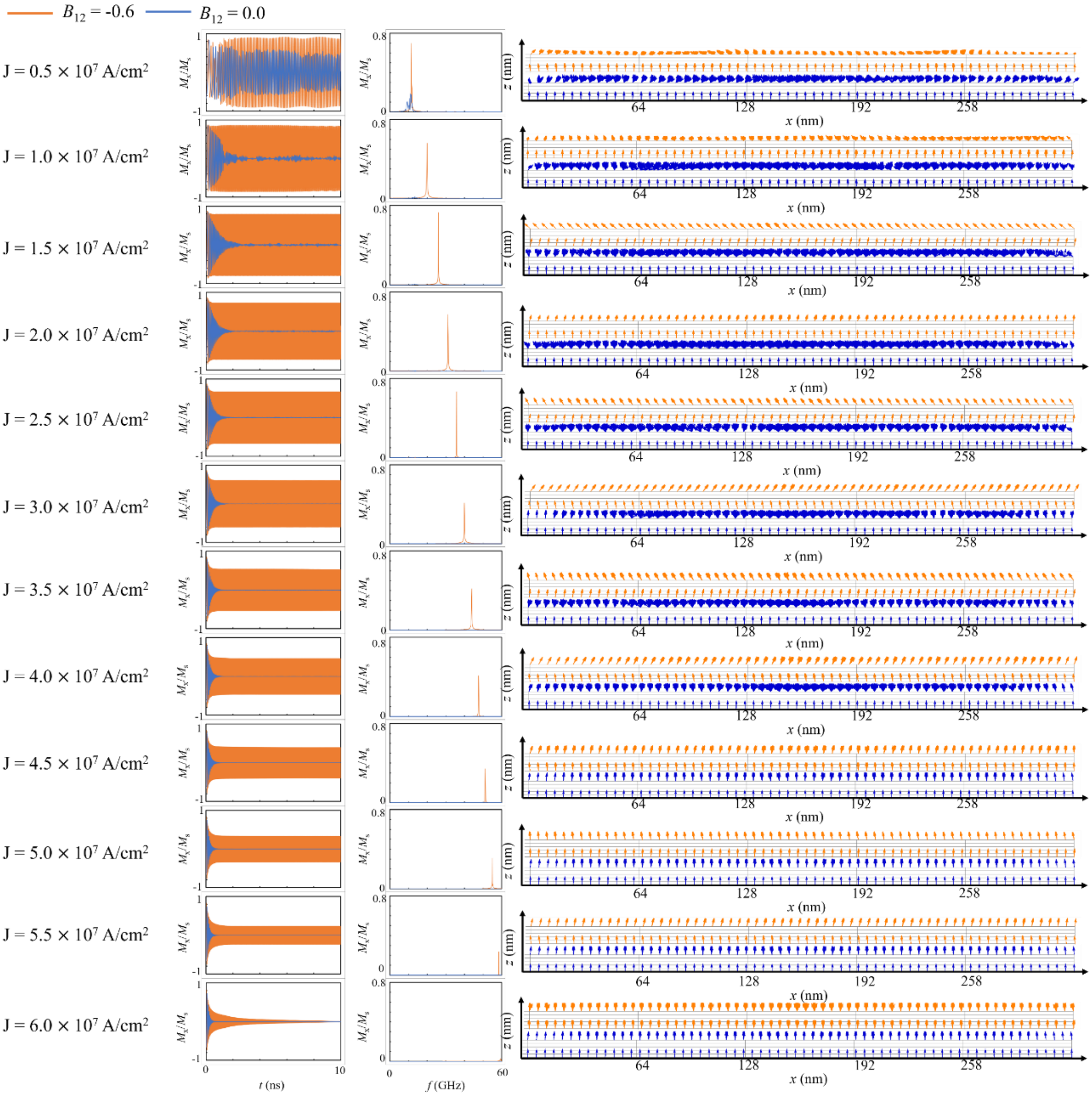

Fig. S6 STO performances for the top layer Ni in the orthogonal configuration where the initial state is in-plane, namely y-axis. The electrical current density was varied from $0.5{\times}10^7$ A/cm^2 to $6.0{\times}10^7$ A/cm^2.

We calculated the top views of STO behaviors changing the current density for the top layer $Co_{90}Fe_{10}$, $Ni_{80}Fe_{20}$, and Ni in the orthogonal configuration where the initial state is out of plane (*z*-axis) and in-plane (*y*-axis). The calculation conditions are shown in Table S2.

Table S2 Calculation conditions in Fig. S7-S12.

Fig.	Top layer	Magnetization in initial state	Current density
S7	$Co_{90}Fe_{10}$	out of plane (*z* axis)	8.0×10^7 A/cm^2 ~ 20.0×10^7 A/cm^2
S8	$Ni_{80}Fe_{20}$	out of plane (*z* axis)	0.5×10^7 A/cm^2 ~ 6.0×10^7 A/cm^2
S9	Ni	out of plane (*z* axis)	0.5×10^7 A/cm^2 ~ 6.0×10^7 A/cm^2
S10	$Co_{90}Fe_{10}$	in-plane (*y* axis)	8.0×10^7 A/cm^2 ~20.0×10^7 A/cm^2
S10	$Ni_{80}Fe_{20}$	in-plane (*y* axis)	0.5×10^7 A/cm^2 ~ 6.0×10^7 A/cm^2
S12	Ni	in-plane (*y* axis)	0.5×10^7 A/cm^2 ~ 6.0×10^7 A/cm^2

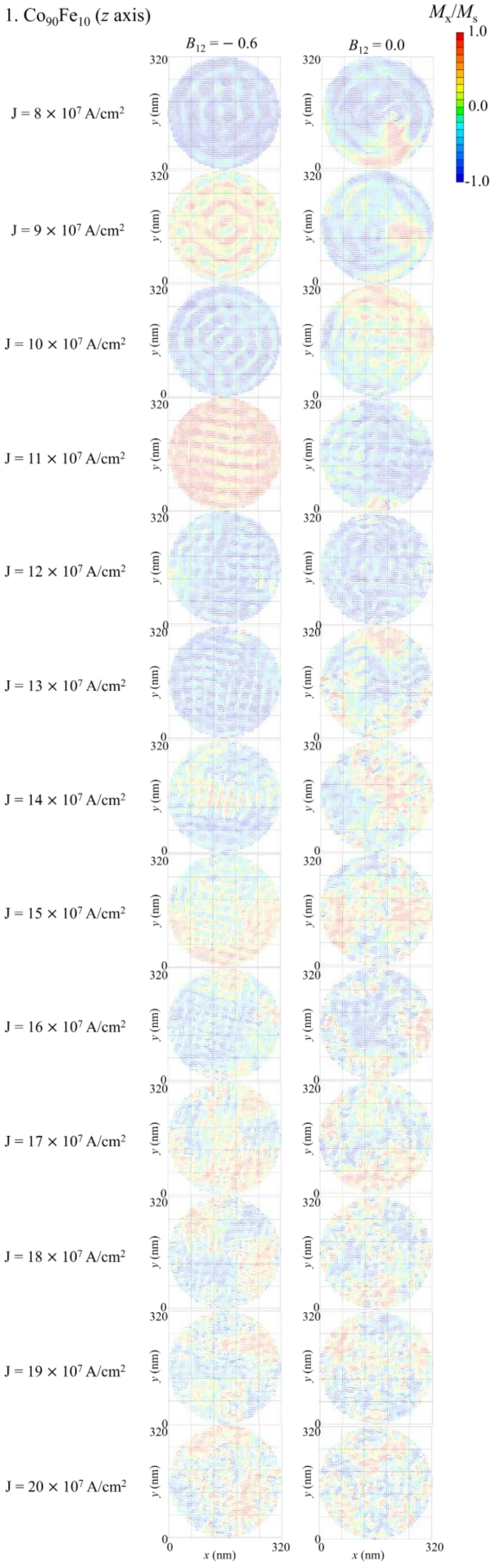

Fig. S7 Top views of STO performances for the top layer $Co_{90}Fe_{10}$ in the orthogonal configuration where the initial state is out of plane, namely z-axis. The electrical current density was varied from 8×10^7 A/cm^2 to 20×10^7 A/cm^2.

2. $Ni_{80}Fe_{20}$ (*z* axis)

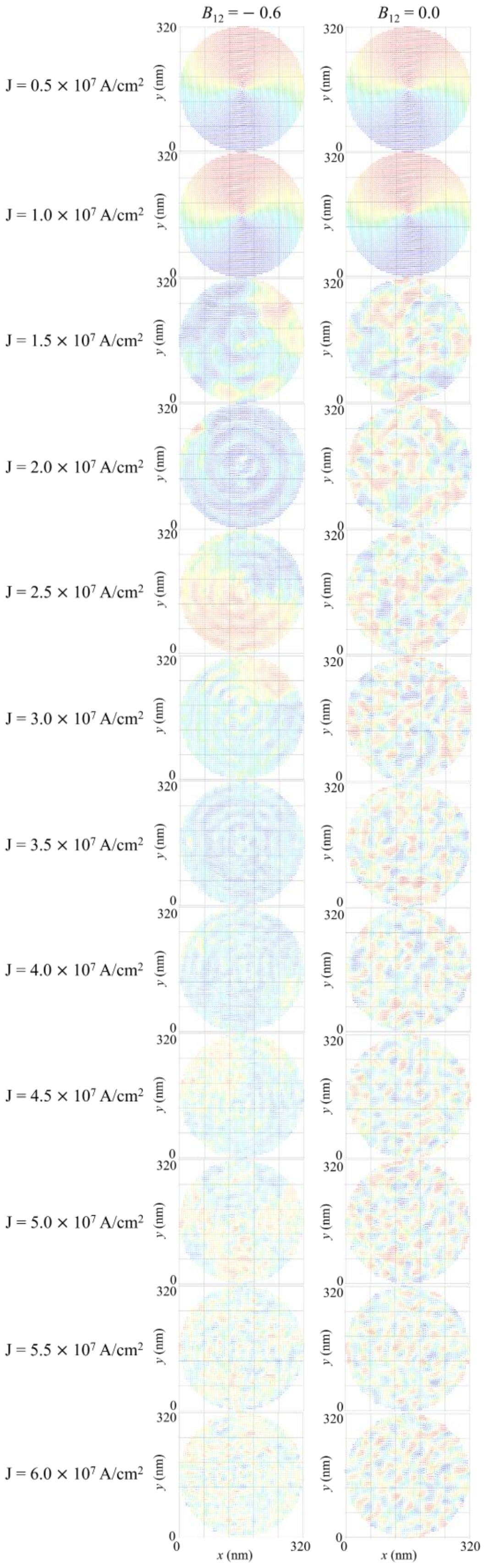

Fig. S8 Top views of STO performances for the top layer $Ni_{80}Fe_{20}$ in the orthogonal configuration where the initial state is out of plane, namely z-axis. The electrical current density was varied from 0.5×10^7 A/cm^2 to 6.0×10^7 A/cm^2.

3. Ni (z axis)

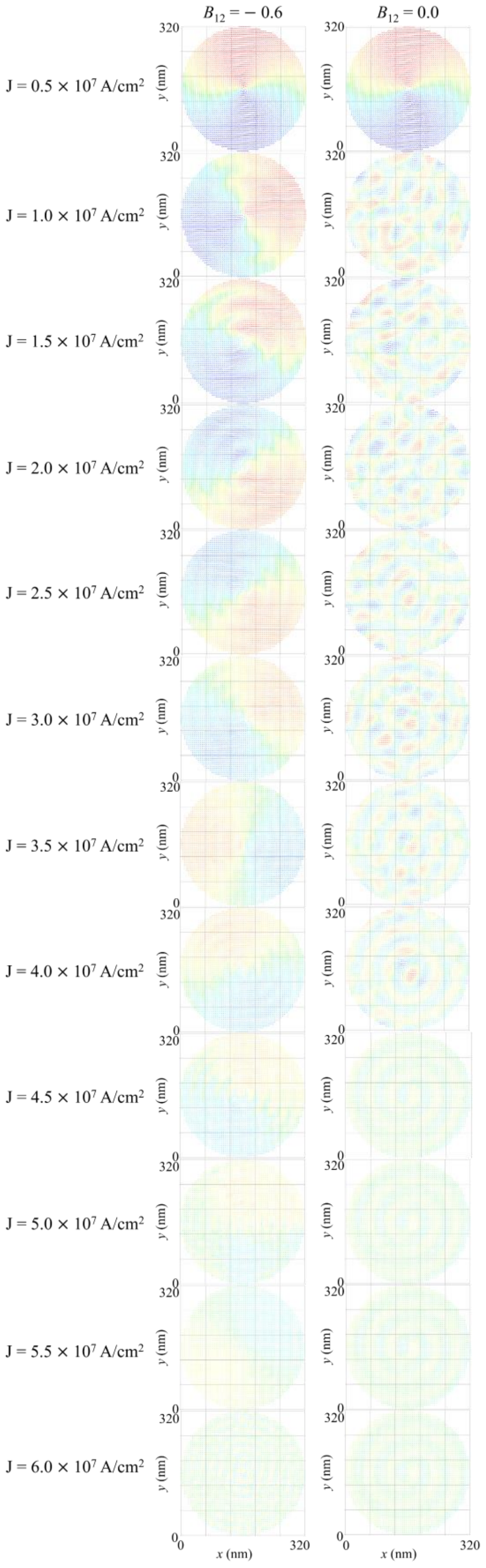

Fig. S9 Top views of STO performances for the top layer Ni in the orthogonal configuration where the initial state is out of plane, namely z-axis. The electrical current density was varied from 0.5×10^7 A/cm^2 to 6.0×10^7 A/cm^2.

4. $Co_{90}Fe_{10}$ (y axis)

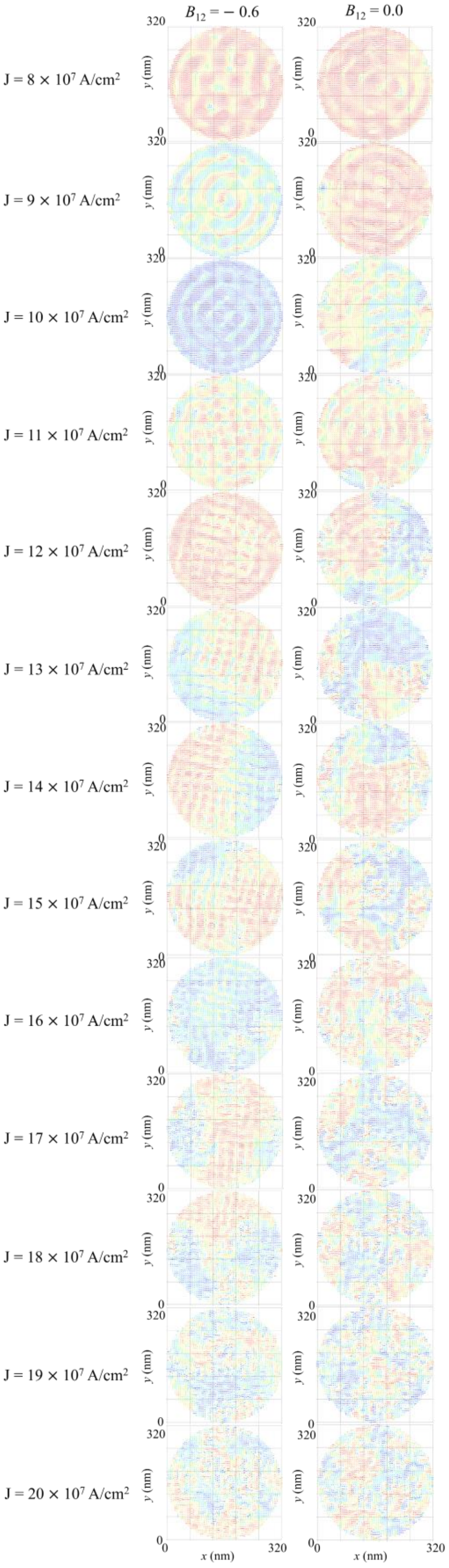

Fig. S10 Top views of STO performances for the top layer $Co_{90}Fe_{10}$ in the orthogonal configuration where the initial state is in-plane, namely y-axis. The electrical current density was varied from 8×10^7 A/cm^2 to 20×10^7 A/cm^2.

5. $Ni_{80}Fe_{20}$ (y axis)

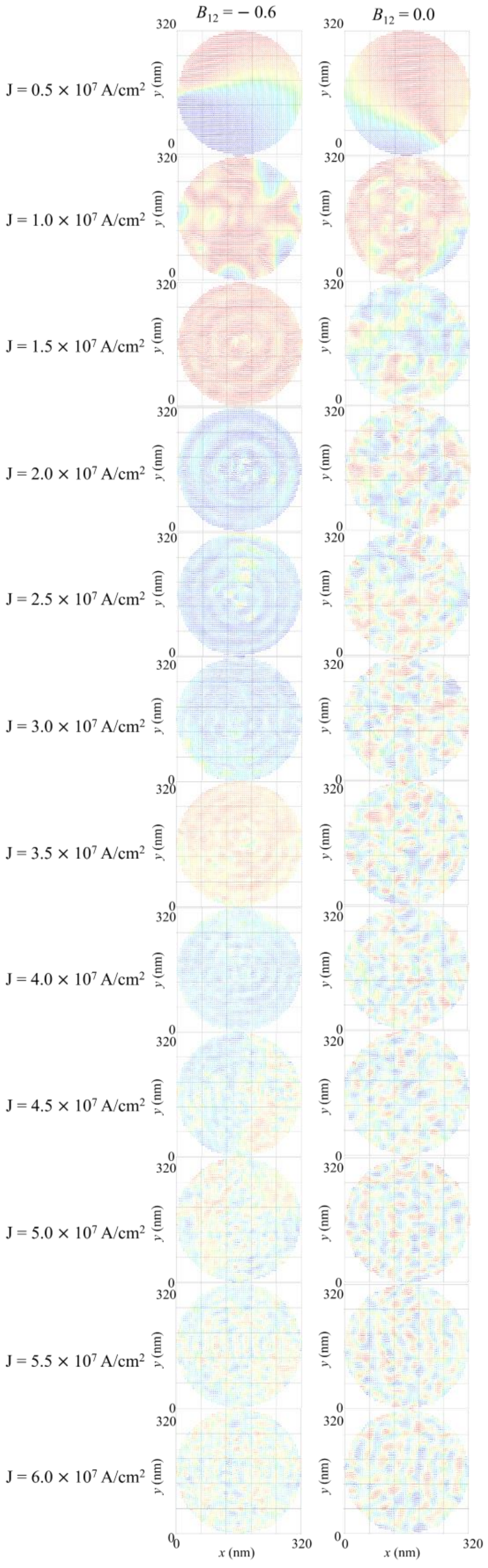

Fig. S11 Top views of STO performances for the top layer $Ni_{80}Fe_{20}$ in the orthogonal configuration where the initial state is in-plane, namely y-axis. The electrical current density was varied from 0.5×10^7 A/cm^2 to 6.0×10^7 A/cm^2.

6. Ni (y axis)

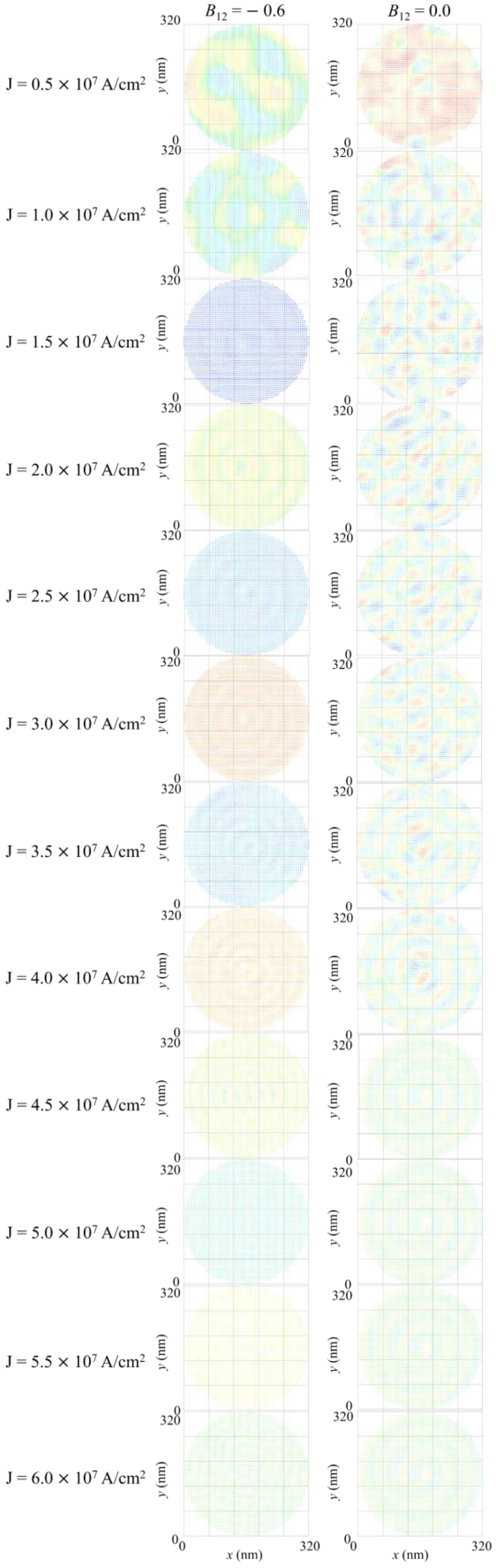

Fig. S12 Top views of STO performances for the top layer Ni in the orthogonal configuration where the initial state is in-plane, namely y-axis. The electrical current density was varied from 0.5×10^7 A/cm^2 to 6.0×10^7 A/cm^2.

We calculated the side views of STO behaviors changing the current density for the top layer $Co_{90}Fe_{10}$, $Ni_{80}Fe_{20}$, and Ni in the orthogonal configuration where the initial state is out of plane (*z*-axis) and in-plane (*y*-axis). The calculation conditions are shown in Table S3.

Table S3 Calculation conditions in Fig. S13-S18.

Fig.	Top layer	Magnetization in initial state	Current density
S13	$Co_{90}Fe_{10}$	out of plane (*z* axis)	8.0×10^7 A/cm^2 ~ 20.0×10^7 A/cm^2
S14	$Ni_{80}Fe_{20}$	out of plane (*z* axis)	0.5×10^7 A/cm^2 ~ 6.0×10^7 A/cm^2
S15	Ni	out of plane (*z* axis)	0.5×10^7 A/cm^2 ~ 6.0×10^7 A/cm^2
S16	$Co_{90}Fe_{10}$	in-plane (*y* axis)	8.0×10^7 A/cm^2 ~20.0×10^7 A/cm^2
S17	$Ni_{80}Fe_{20}$	in-plane (*y* axis)	0.5×10^7 A/cm^2 ~ 6.0×10^7 A/cm^2
S18	Ni	in-plane (*y* axis)	0.5×10^7 A/cm^2 ~ 6.0×10^7 A/cm^2

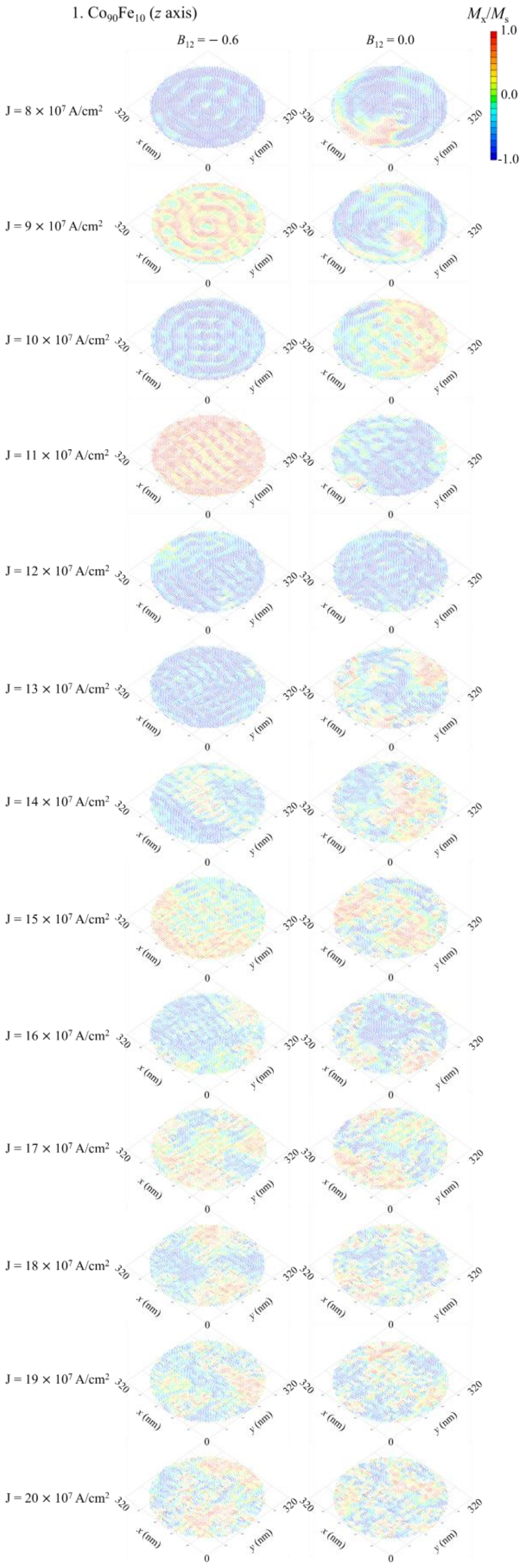

Fig. S13 Side views of STO performances for the top layer $Co_{90}Fe_{10}$ in the orthogonal configuration where the initial state is out of plane, namely z-axis. The electrical current density was varied from 8×10^7 A/cm^2 to 20×10^7 A/cm^2.

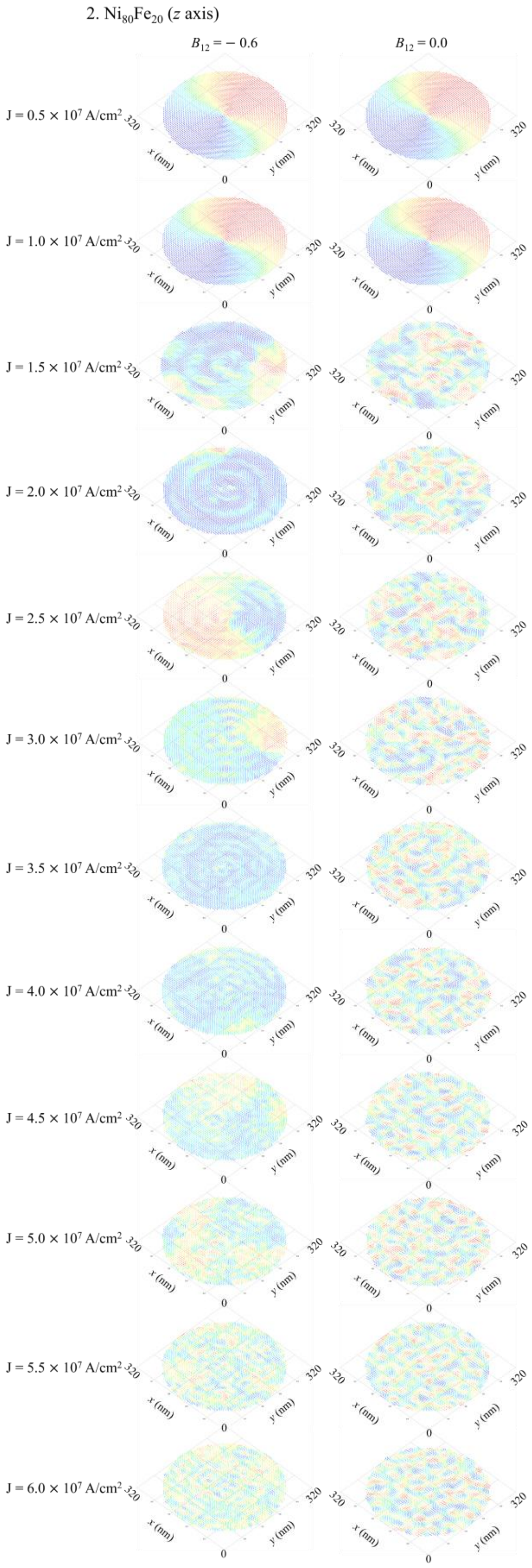

Fig. S14 Side views of STO performances for the top layer $Ni_{80}Fe_{20}$ in the orthogonal configuration where the initial state is out of plane, namely z-axis. The electrical current density was varied from 0.5×10^7 A/cm^2 to 6.0×10^7 A/cm^2.

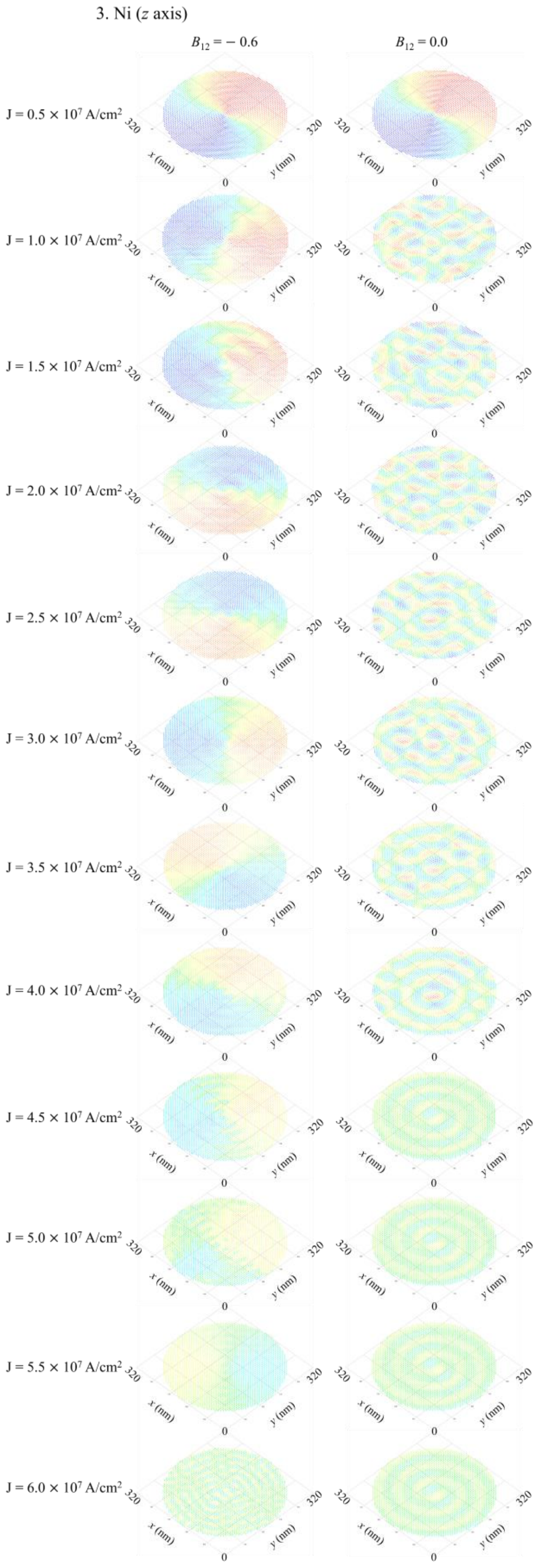

Fig. S15 Side views of STO performances for the top layer Ni in the orthogonal configuration where the initial state is out of plane, namely z-axis. The electrical current density was varied from 0.5×10^{7} A/cm^{2} to 6.0×10^{7} A/cm^{2}.

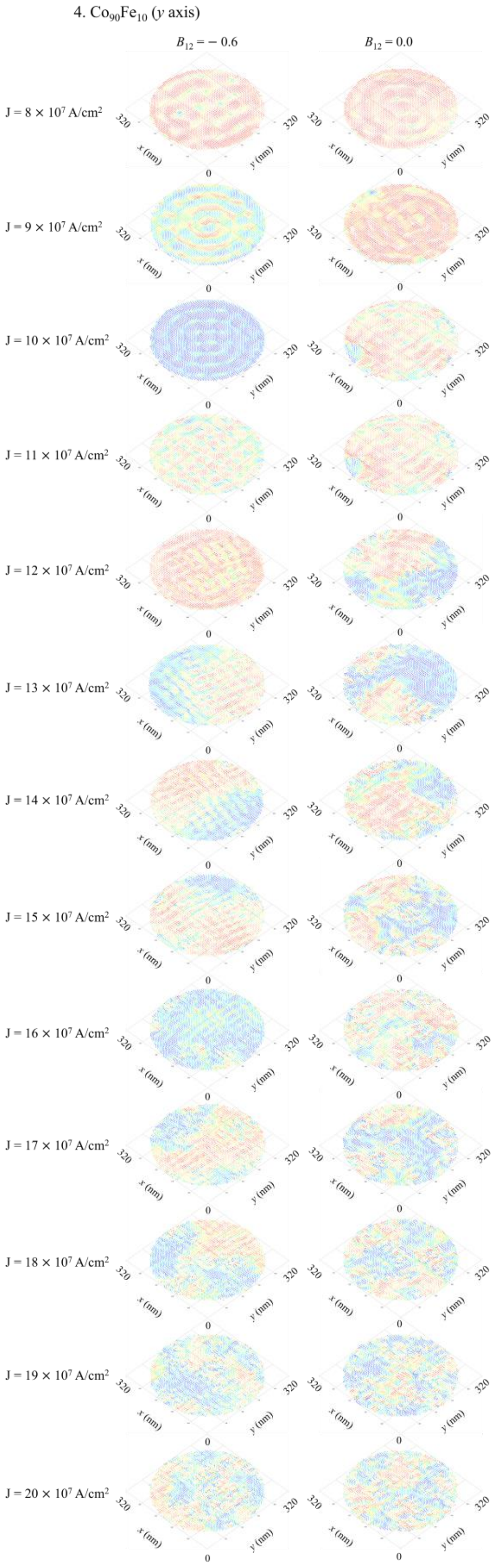

Fig. S16 Side views of STO performances for the top layer $Co_{90}Fe_{10}$ in the orthogonal configuration where the initial state is in-plane, namely y-axis. The electrical current density was varied from 8×10^7 A/cm^2 to 20×10^7 A/cm^2.

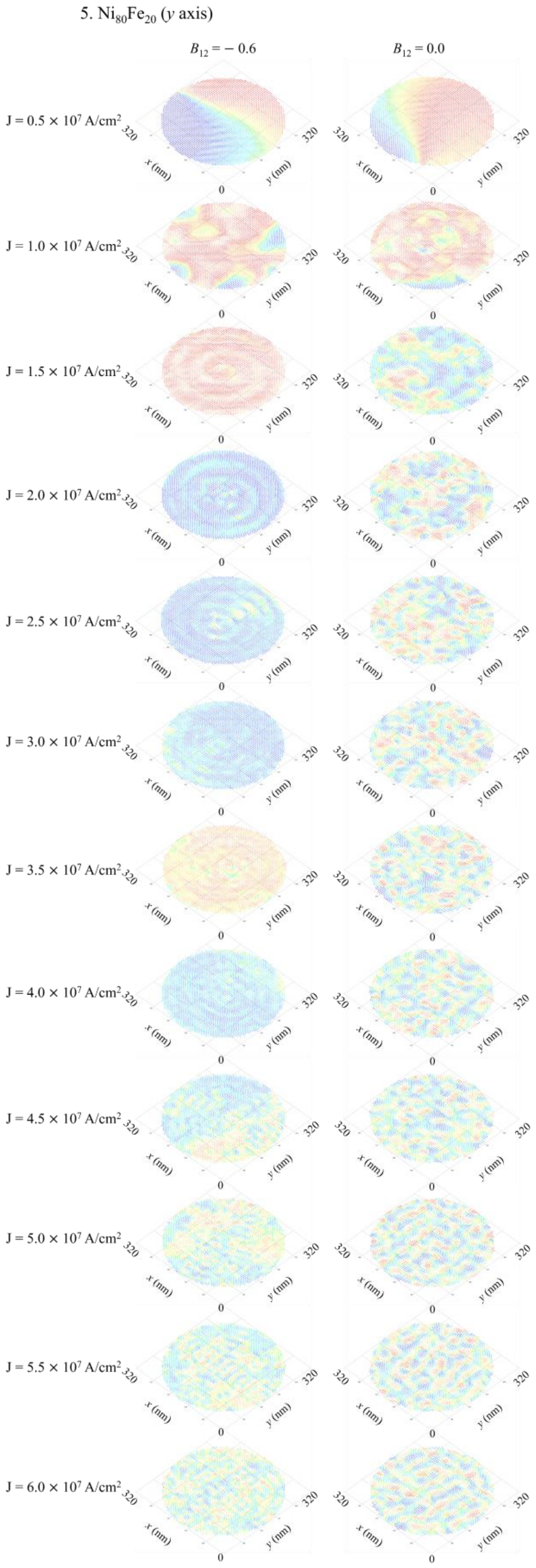

Fig. S17 Side views of STO performances for the top layer $Ni_{80}Fe_{20}$ in the orthogonal configuration where the initial state is in-plane, namely y-axis. The electrical current density was varied from 0.5×10^7 A/cm^2 to 6.0×10^7 A/cm^2.

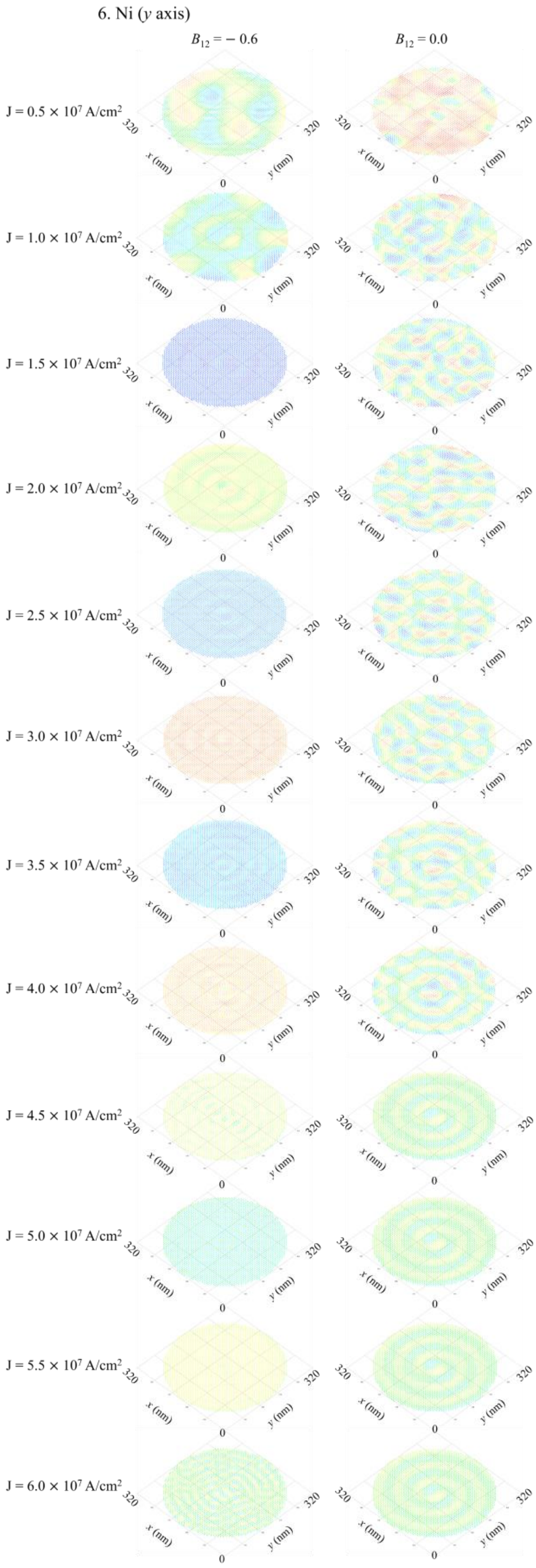

Fig. S18 Side views of STO performances for the top layer Ni in the orthogonal configuration where the initial state is in-plane, namely y-axis. The electrical current density was varied from 0.5×10^7 A/cm^2 to 6.0×10^7 A/cm^2.

2. Intensity fluctuation against current density

In Fig. 6(b), M_x intensity is not monotone against the current density. The main reason of the M_x intensity fluctuation is considered to be the spin wave. The generation of spin wave depends on the device size/shape and current density. Let us consider the M_x intensity jump from small, large, and small value of Ni top layer with B_{12}=−0.6 by changing the current density from 1.0 ×10^7 A/cm^2, 1.5 ×10^7 A/cm^2, to 2.0 ×10^7 A/cm^2 in Fig. 6(b). Figures S7 show the spin waves under three kinds of current density. The spin wave under 1.0 ×10^7 A/cm^2 and 2.0 ×10^7 A/cm^2 (Fig. S7(a) and (c)) are larger than that under 1.5 ×10^7 A/cm^2 (Fig. S7(b)). It was found that when the spin wave is weak, the M_x shows the high intensity. Since the spin wave effect diminishes as increasing current density, the M_x intensity fluctuation is apparent in the low current region in Fig. 6(b).

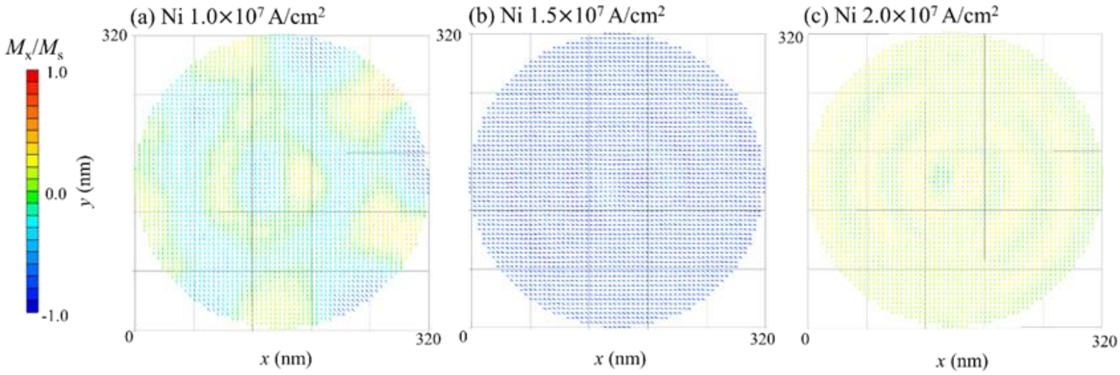

Fig. S19 Top views for Ni top layer after 10 ns during oscillation after y-axis initialization when the current density is 1.0×10^7 A/cm^2 (a), 1.5×10^7 A/cm^2 (b), and 2.0×10^7 A/cm^2 (c).

3. Angles from *z*-axis

We estimated the angles of magnetization from *z*-axis in each cell for the Ni top layer at 10 ns after the start of the current flow with 1.5×10^7 A/cm^2, as shown in Fig. S20. The angles of magnetizations shown in Fig. 5 (c)(vii) were averaged for the y-direction. The angle variation at $B_{12} = -0.6$ is smaller than that at $B_{12} = 0.0$, leading the sable STO. The average of angle at $B_{12} = -0.6$ is larger than that at $B_{12} = 0.0$, indicating that the magnetization reversal can be suppressed by introducing biquadratic magnetic coupling to widen current density region.

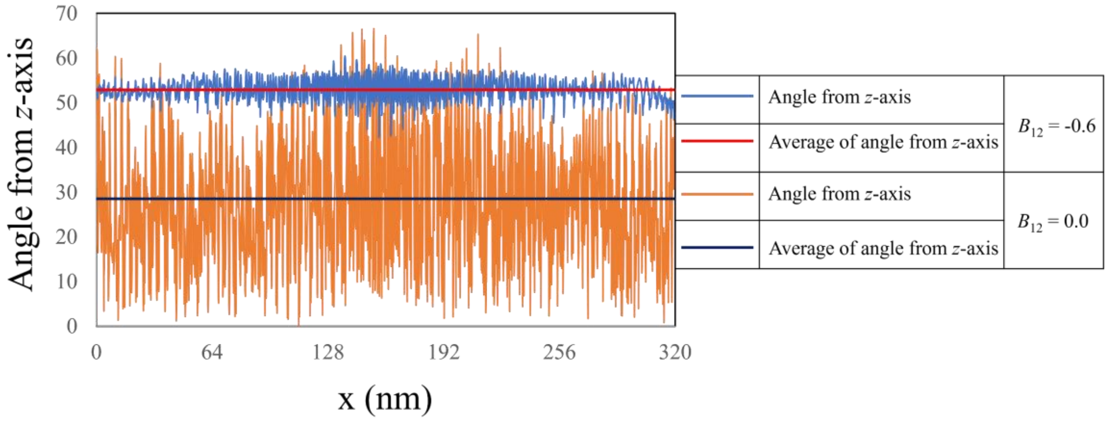

Fig. S20 Angle of magnetization in each cell from *z*-axis at 10 ns after starting current flow for the Ni top layer under 1.5×10^7 A/cm^2. Blue and orange lines denote the angle in the cells averaged for y-direction, red and black lines denote the angle further averaged for x-direction for $B_{12} = -0.6$ and $B_{12} = 0.0$, respectively.
