# Supplementary material for: High-frequency spin torque oscillation in orthogonal magnetization disks with strong biquadratic magnetic coupling
Source: Sci Rep. 2023 Mar 3;13:3631. doi: 10.1038/s41598-023-30838-y (PMC9984381; doi:10.1038/s41598-023-30838-y)
Supplement: Supplementary file 4 — Supplementary Information 2. [file 41598_2023_30838_MOESM4_ESM.pdf]

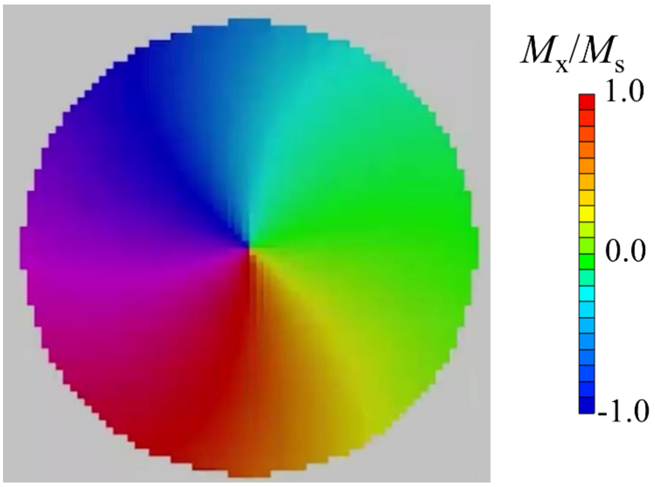

Suppl 2. Magnetic moment dynamic video for the top layer of Ni in the orthogonal configuration with an out-of-plane initial state, namely the *z*-axis. The $B_{12} = -0.6$. The electrical current density was 3.0×10^7 A/cm^2.

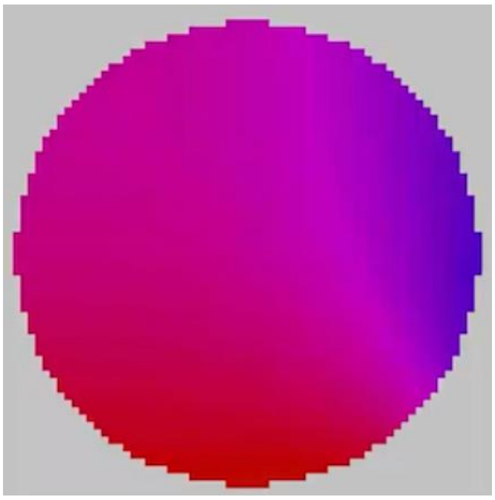

Suppl 3. Magnetic moment dynamic video for the top layer of Ni in the orthogonal configuration with an out-of-plane initial state, namely the *y*-axis. The $B_{12} = -0.6$. The electrical current density was 3.0×10^7 A/cm^2.
